# Clinical Outcome and Medical Cost of Originator and Generic Antihypertensive Drugs: A Population-Based Study in Yinzhou, China

**DOI:** 10.3389/fphar.2022.757398

**Published:** 2022-02-22

**Authors:** Tao Huang, Lin Bai, Haishaerjiang Wushouer, Zhiyuan Wang, Mingchun Yang, Hongbo Lin, Peng Shen, Xiaodong Guan, Luwen Shi

**Affiliations:** ^1^ Department of Pharmacy Administration and Clinical Pharmacy, School of Pharmaceutical Sciences, Peking University, Beijing, China; ^2^ International Research Center for Medicinal Administration, Peking University, Beijing, China; ^3^ Yinzhou District Center for Disease Control and Prevention, Ningbo, China

**Keywords:** clinical outcome, generic, comparative effectiveness research, antihypertenisve, originator

## Abstract

**Background:** The substitution of generic drugs can effectively alleviate the rapid growth of drug costs; however, the clinical effectiveness and medical costs of originator products and generics were barely studied in China.

**Objectives:** To compare the effectiveness of antihypertensive drugs and hypertension-related medical costs between originator and generic initiators in Yinzhou, China.

**Methods:** We conducted a population-based retrospective cohort study using the Chinese Electronic Health Records Research in Yinzhou (CHERRY), from July 1, 2011, to December 31, 2018. Hypertension patients initiating with originator products were compared with patients initiating with generic counterparts. We used 1:1 propensity score matching to pair the two groups based on sociodemographic, clinical, and health service utilization variables. Cox proportional regression was adopted to compare the rate of hospitalization for hypertension-related cardiovascular disease between matched originator and generic initiators. Wilcoxon matched-pairs signed-rank test was used to compare annual hypertension-related medical costs.

**Results:** Matched pairs (10,535) of patients were included in the comparative study of originator products and generics, corresponding to seven antihypertensive drugs including amlodipine, felodipine, nifedipine, irbesartan, losartan, valsartan, and metoprolol. The average age of patients included in the analysis was around 60 years (originator *vs*. generics initiators: from 59.0 *vs*. 59.1 years in losartan to 62.9 *vs*. 63.6 years in nifedipine). Higher hospitalization rates among originator initiators were observed for three calcium channel blockers (hazard ratio[95% CI]: amlodipine, 3.18[1.43, 7.11]; felodipine, 3.60[1.63, 7.98]; and nifedipine, 3.86[1.26, 11.81]; respectively). The remaining four out of seven drugs of the clinical endpoint estimates showed comparable outcomes between originator products and generics (hazard ratio[95% CI]: irbesartan, 1.19[0.50, 2.84]; losartan, 1.84[0.84, 4.07]; valsartan, 2.04[0.72, 5.78]; and metoprolol, 1.25[0.56, 2.80]; respectively). Higher median annual hypertension-related medical costs were observed in originator initiators (all *p* < 0.001), except for metoprolol (*p* = 0.646).

**Conclusion:** We observed comparable or even better clinical outcomes and less medical cost associated with the use of antihypertensive generics compared to originator counterparts. This could help increase patient and provider confidence in the efficacy of generic medicines to manage hypertension diseases.

## Introduction

Increasing drug cost has emerged as a critical public health issue, straining the financial budgets of patients and contributing to poor medication adherence or treatment discontinuation (Su et al., 2017; Husain et al., 2020). Originator products sold at high prices have been a major contributor to elevated drug costs (Haas et al., 2005; Kesselheim et al., 2008). Thus, many countries, including the United States, Canada, the Netherlands, and some other European countries (Shrank et al., 2010; Godman et al., 2014; Mishuk et al., 2020; Godman et al., 2021), promoted substituting originators with less expensive generic drugs to control health expenditures and improve medication adherence (Shrank et al., 2006; WHO, 2010; Dylst and Simoens, 2011; Godman et al., 2014; Godman et al., 2021).

Generics are approved based on evidence of pharmaceutical equivalence and bioequivalence with originator drugs. Several systematic reviews and meta-analyses have compared the clinical characteristics of generics and originator products used for cardiovascular diseases (CVD) and showed no superiority of the latter over the former. Nonetheless, heterogeneities remained between studies, and most studies included were bioequivalence trials (Kesselheim et al., 2008; Manzoli et al., 2016; Leclerc et al., 2020). Although several observational studies have investigated the clinical equivalence of generics to originator products, they demonstrated ambiguous results (Kesselheim et al., 2008; Manzoli et al., 2016; Desai et al., 2019; Leclerc et al., 2020). Given a lack of real-world evidence, many patients still perceived generics as less clinically effective and safe with the belief that being cheap implied being inferior (Babar et al., 2011; Ngo et al., 2013; Dunne and Dunne, 2015; Toverud et al., 2015).

In China, the government has implemented a series of health policies to encourage the research and development of generics to promote market competition and reduce drug costs. However, bioequivalence studies are optional in the approval of generics in China. A lack of bioequivalence results in undermining the confidence of both health professionals and patients in the clinical effectiveness of generics, contributing to a relatively low prescribing rate of generics in China (Zeng, 2013; Huang et al., 2017; Jiang et al., 2020). Therefore, a better understanding of the comparative effectiveness of generics and their originator counterparts is urgently needed. Using a population-based data of Yinzhou, this study aimed to compare the clinical outcome and hypertension-related medical costs between patients initiating originator and generic antihypertensive drugs and to contribute to the evidence for better clinical decision-making.

## Materials and Methods

### Study Design and Data Source

We conducted a population-based retrospective cohort study using the Chinese Electronic Health Records Research in Yinzhou (CHERRY) from July 1, 2011, to December 31, 2018.

The CHERRY was a relational database, including different administrative databases of sociodemographic characteristics, health check and death surveillance data, patient electronic medical records, and health insurance information. Since 2009, the CHERRY has covered 98% of permanent residents (about 1.24 million) in Yinzhou, Ningbo, Zhejiang. Details about the database could be found in previous studies (Lin et al., 2018; Yang et al., 2018). We extracted the following variables from the database in this study: 1) patient sociodemographic characteristics including sex, age, and insurance type; 2) prescription data including drug trade name, international nonproprietary name (INN), drug code (Anatomical Therapeutic Chemical Classification of Medications, ATC code), prescription date, and usage; 3) patient clinical information including diagnosis names, diagnosis type, diagnosis code (International Classification of Diseases, Tenth Revision, and ICD-10 code) and diagnosis date; and 4) patient death date from health check and death surveillance database.

### Study Population and Follow-Up

We included patients aged ≥18 years who were diagnosed with hypertension (ICD-10 code: I10-I15) between July 1, 2011, and December 31, 2018, in the CHERRY database. The first antihypertensive drug prescription of each patient was identified as the index prescription, and the corresponding date was regarded as the index date. We used 90 days for the induction period (minimal time needed between drug initiation and disease occurrence) and 0 days for the latent period (maximal time between drug modification and disease occurrence) (Lund et al., 2015). All patients included were followed from index date until the occurrence of the following events, whichever came first: 1) primary outcome, defined as hospitalization with hypertension-related CVD; 2) treatment discontinuation, defined as over 90 days lag time following the last dispensing; 3) treatment modification, including adding or transferring to another antihypertensive drug, 4) treatment switch, defined as switching from generics to originator counterparts or vice versa according to the originator manufacturer information on the National Medical Products Administration (NMPA) website (National Medical Products Administration, 2020); 5) death; and 6) end of the study (December 31, 2018).

**FIGURE 1:**
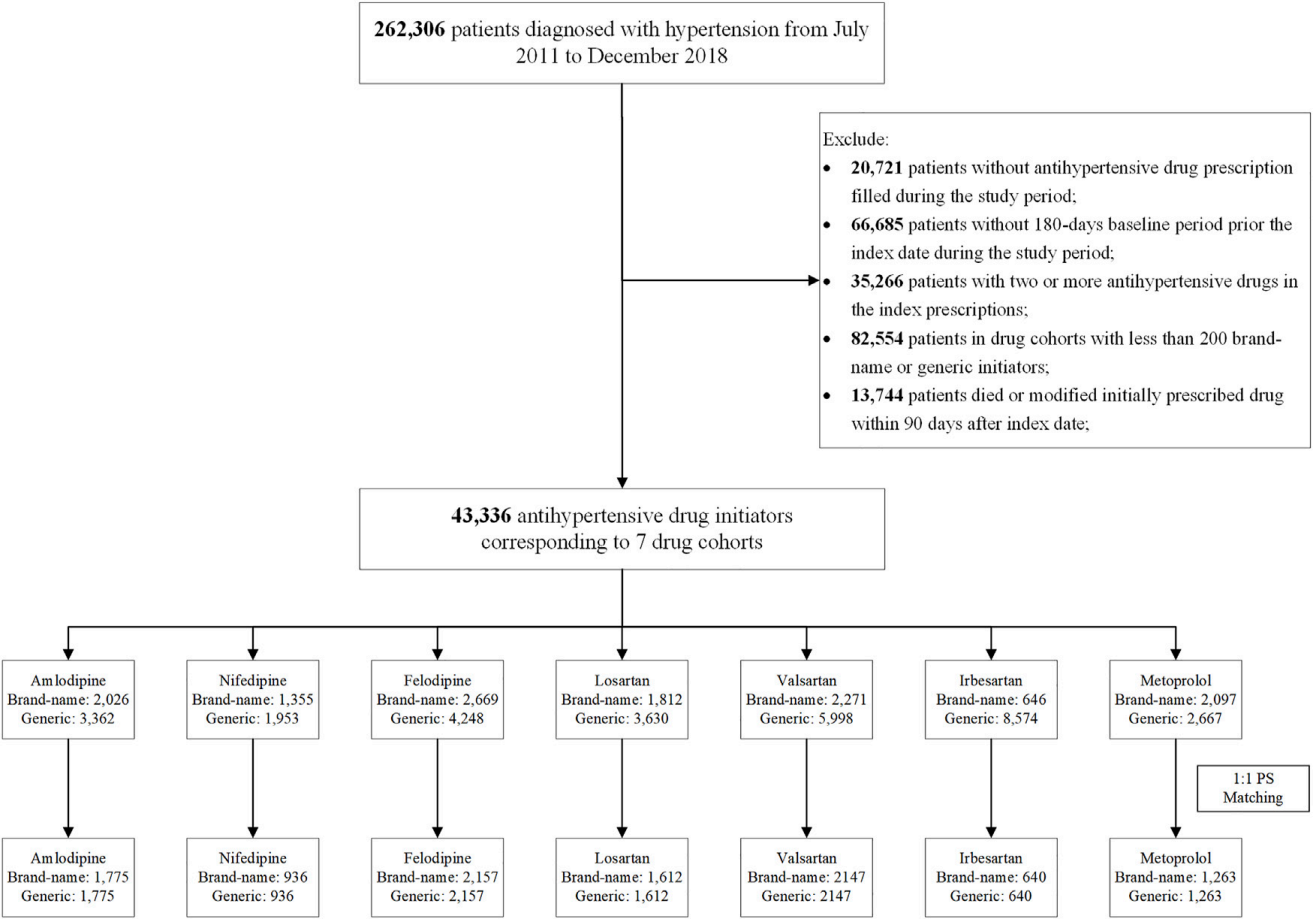
Flow of sample selection.

We excluded the following: 1) patients without antihypertensive drug (details of drug information are in Supplementary Table S1) prescription filled during the study period; 2) patients without 180-day baseline period prior to the index date during the study period; 3) patients who initiated two or more antihypertensive drugs in the index prescription; and 4) patients who died or modified their initial antihypertensive drugs within 90 days after the index date (Figure 1).

Then we divided the patients into different study cohorts according to the INNs of their initially prescribed antihypertension drugs (e.g., amlodipine cohort, losartan cohort). In each drug cohort, patients were subsequently classified into either originator or generic initiators based on the originator manufacturer information from NMPA website (National Medical Products Administration, 2020). To obtain sufficient observed outcome events, we excluded patients with less than 200 originators or generic initiators (Figure 1).

### Outcomes

The primary outcome was hospitalization with hypertension-related CVD, identified by the primary discharge diagnosis of patients (ICD-10 code I00-I25, I27-I88, and I95-I99) (Lewington et al., 2016).

The annual hypertension-related medical cost [in renminbi (RMB)] for each patient was calculated as total hypertension-related medical cost of outpatient visits during the follow-up period, including medication costs and examination costs (identified by the outpatient diagnosis (ICD-10 code: I10-I15), divided by the number of followed years.

### Covariates

The main independent variable of interest was the generic or originator antihypertensive drug prescribed at the index date. Covariates were measured during the 180-day baseline period, including the following: 1) sociodemographic characteristics, including sex, age at the index date, and insurance type; 2) drug use information, comprising statins and other lipid lowering drugs, antiplatelets, insulin preparations, oral hypoglycemic agents, aspirin, other nonsteroidal anti-inflammatory drugs (NSAIDs), nitrates, anticoagulants, digoxin, antiarrhythmics, and Coxibs (Supplementary Table S2); 3) health service utilization variables, containing all-cause outpatient visits, all-cause emergency department (ED) visits, and inpatient visits; and 4) the Charlson comorbidity index (CCI) score, estimated according to the baseline clinical information (Sundararajan et al., 2007).

### Statistical Analysis

Within each drug cohort, propensity score was calculated by fitting a logistic regression model to predict the probability of initiating originator products *vs*. generics, as a function of the baseline covariates. A 1:1 propensity score matching using greedy nearest neighbor caliper matching without replacement was performed to balance the confounders between originator and generic initiators. A caliper width of 0.2 of the standard difference of the logit of the propensity score was used (Austin, 2011). Standardized mean differences (SMD) were used to estimate the differences of the covariates before and after matching between the two groups. A SMD <0.1 was considered to be statistically negligible (Normand et al., 2001).

In the matched cohort, the incidence rate was calculated, and the crude hazard ratio (HR) of hospitalization with hypertension-related CVD between originator *vs*. generic initiators was estimated by using Cox proportional hazard regression model with a robust sandwich-type variance estimator to account for the matched nature of the sample (Lin and Wei, 1989; Austin, 2013). Furthermore, the crude hazard ratio for treatment discontinuation, treatment switch, and treatment modification of originator *vs*. generic initiators were estimated. The proportional hazards assumption was assessed by the Schoenfeld residuals test. Annual hypertension-related medical costs were calculated and compared using either matched *t*-test or Wilcox matched-pairs signed-rank test between two groups.

All analyses were performed using the Stata (version 14.1). Ninety-five percent confidence interval (CI) and *p*-value were reported. A two-side *p*-value <0.05 was considered to be statistically significant.

### Sensitive Analysis

We conducted the following sensitivity analyses to test the robustness of our results. First, subgroup analyses were performed to test the potential effect modification of age; patients without prior hospitalization; emergency visits in the baseline period; patients without prior diagnosed myocardial infarction (MI), stroke, or congestive heart failure (CHF) in the baseline period; and patients without treatment discontinuation within the early 180 days in the follow-up period, respectively. Second, as the mechanism for hypertension-inducing CVD is unclear, different induction and latent time intervals (0, 30, 60, and 90 days) were used to compare the results.

### Ethics Statement

The research was granted ethical exemption by the Ethical Committee of Peking University (No.208027). Participants were not involved in the study design, data extraction, and analysis.

## Results

### Patient Characteristics

A total of 43,336 hypertension patients were included in the comparisons of originator and generic initiators. After propensity score matching, 21,070 patients remained across seven drugs (amlodipine, felodipine, nifedipine, irbesartan, losartan, valsartan, and metoprolol) (Figure 1).

Baseline characteristics of patients in each drug cohort between originator and generic initiators were summarized in Supplementary Tables S3–S9. After propensity score matching, baseline variables were balanced between the two groups except index year of nifedipine and felodipine, and aspirin use in losartan. The study population aged around 60 years (originator *vs*. generics initiators: ranged from 59.0 *vs*. 59.1 years in losartan to 62.9 *vs*. 63.6 years in nifedipine). More patients were enrolled in the medical insurance for urban employees (UEBMI) or medical insurance for urban residents (URBMI) (originator *vs*. generic initiators: ranged from 65.1% *vs*. 63.8% in metoprolol to 93.6% *vs*. 92.2% in irbesartan). The average baseline CCI score was about 0.3 (originator *vs*. generic initiators: ranged from 0.28 *vs*. 0.25 in amlodipine to 0.40 *vs*. 0.45 in irbesartan).

### Hospitalization for Hypertension-Related CVD

The median follow-up time for originator initiators ranged from 0.30[IQR:0.25, 0.77] years in metoprolol to 0.48[IQR: 0.25, 1.21] years in irbesartan and valsartan, and that of the generic initiators ranged from 0.44[IQR: 0.25, 1.04] years in metoprolol to 0.70[IQR: 0.34, 1.47] years in irbesartan. Higher hospitalization rates in the originator initiators were observed for the three calcium channel blockers (CCB) (HR [95% CI]: amlodipine, 3.18[1.43, 7.11]; felodipine, 3.60[1.63, 7.98]; and nifedipine, 3.86[1.26, 11.81]; respectively) (Table 1). For angiotensin receptor blockers (ARBs) and beta-blockers, no significant differences were found in the hospitalization rates for hypertension-related CVD between originator initiators *vs*. generic initiators (HR [95% CI]: irbesartan, 1.19[0.50, 2.84]; losartan, 1.84[0.84, 4.07]; valsartan, 2.04[0.72, 5.78]; and metoprolol, 1.25[0.56, 2.80]; respectively) (Table 1).

**TABLE 1:** Hospitalization for hypertension-related CVD of originator *vs*. generic initiators after 1:1 propensity score matching.

Drug		Group	Sample size, n	Follow-up, median (IQR)/years	Total person-years	Hospitalization events, n	Hospitalization rate/1,000 person-years	HR (95% CI)
CCBs	Amlodipine	Originator	1,775	0.47 (0.25, 1.15)	1,640	23	14	3.18 (1.43, 7.11)
		Generic	1,775	0.61 (0.30, 1.34)	1,710	7	4.1	Reference
	Felodipine	Originator	2,157	0.38 (0.25, 0.95)	1,906	24	12.6	3.60 (1.63, 7.98)
		Generic	2,157	0.51 (0.25, 1.25)	2,171	9	4.15	Reference
	Nifedipine	Originator	936	0.38 (0.25, 0.92)	840	16	19	3.86 (1.26, 11.81)
		Generic	936	0.46 (0.25, 1.05)	786	4	6	Reference
ARBs	Irbesartan	Originator	645	0.48 (0.25, 1.21)	584	11	18.8	1.19 (0.50, 2.84)
		Generic	645	0.70 (0.34, 1.47)	643	7	10.9	Reference
	Losartan	Originator	1,612	0.43 (0.25, 0.96)	1,389	16	11.5	1.85 (0.84, 4.07)
		Generic	1,612	0.51 (0.25, 1.17)	1,468	11	7.5	Reference
	Valsartan	Originator	2,147	0.48 (0.25, 1.21)	2,060	10	4.9	2.04 (0.72, 5.78)
		Generic	2,147	0.62 (0.28, 1.38)	2,170	6	2.8	Reference
Beta-blocker	Metoprolol	Originator	1,263	0.30 (0.25, 0.77)	1,083	12	11.1	1.25 (0.56, 2.80)
		Generic	1,263	0.44 (0.25, 1.04)	1,138	11	9.7	Reference

Abbreviations: CVD, cardiovascular diseases; IQR, interquartile range; HR, hazard ratio; CI, confidence interval. CCB, calcium channel blocker; ARB, angiotensin receptor blocker.

### Annual Hypertension-Related Medical Cost

The median annual hypertension costs for originator initiators ranged from RMB715.4 (interquartile range/IQR: 262.8, 1,529.4) for metoprolol to RMB1,595.1 (IQR: 814.0, 2,814.2) for losartan, while the median annual hypertension costs for generic initiators ranged from RMB419.8 (IQR: 171.6, 985.5) for nifedipine to RMB1,204.5 (IQR: 598.6, 2,182.7) for losartan. Higher median annual hypertension-related medical costs were observed in originator initiators (*p* < 0.001), except metoprolol (*p* = 0.646) (Table 2).

**TABLE 2:** Annual hypertension-related medical cost for originator *vs*. generic initiators after 1:1 propensity score matching.

Drug		Group	Sample size, n (missing)^a^	Annual cost, median (IQR)/RMB	*p*-value
CCBs	Amlodipine	Originator	1,775 (166)	1,306.7 (631.5, 2,274.0)	<0.001
		Generic	1,775 (183)	759.2 (350.4, 1,449.1)	
	Felodipine	Originator	2,157 (102)	981.9 (459.9, 1,759.3)	<0.001
		Generic	2,157 (98)	569.4 (262.8, 1,109.6)	
	Nifedipine	Originator	936 (53)	1,259.3 (573.1, 2,332.4)	<0.001
		Generic	936 (41)	419.8 (171.6, 985.5)	
ARBs	Irbesartan	Originator	645 (70)	1,471.0 (704.5, 2,799.6)	<0.001
		Generic	645 (88)	835.9 (390.6, 1,686.3)	
	Losartan	Originator	1,612 (152)	1,595.1 (814.0, 2,814.2)	<0.001
		Generic	1,612 (93)	1,204.5 (598.6, 2,182.7)	
	Valsartan	Originator	2,147 (223)	1,416.2 (737.3, 2,430.9)	<0.001
		Generic	2,147 (128)	861.4 (438.0, 1,584.1)	
Beta-blocker	Metoprolol	Originator	1,263 (33)	704.5 (262.8, 1,529.4)	0.646
		Generic	1,263 (15)	741.0 (266.5, 1,646.2)	

a Patients with missing cost data in the matched cohorts were excluded when comparing hypertension-related medical costs.

Abbreviations: IQR, interquartile range; CCB, calcium channel blocker; ARB, angiotensin receptor blocker.

### Treatment Discontinuation, Switch, and Modification

Higher treatment discontinuation rates were observed in originator initiators in six drugs (HR [95% CI]: amlodipine, 1.28[1.17, 1.39]; felodipine, 1.23[1.14, 1.32]; irbesartan, 1.20[1.04, 1.39]; losartan, 1.31[1.20, 1.43]; valsartan, 1.09[1.01, 1.18]; and metoprolol, 1.29[1.18, 1.40]) except nifedipine (HR [95% CI]: 1.04[0.93, 1.15]) (Table 3). Originator initiators of irbesartan and losartan (HR [95% CI]: 5.50[2.07, 14.65] and 1.95[1.22, 3.13], respectively) were more likely to switch their treatments compared to generic initiators (Table 3). Meanwhile, higher modification rate was observed in originator initiators of metoprolol (HR [95% CI]: 1.28[1.01, 1.60]), and lower modification rates were found in originator initiators of amlodipine and losartan (HR [95% CI]: 0.74[0.63, 0.86] and 0.76[0.64, 0.90], respectively) (Table 3).

**TABLE 3:** Treatment discontinuation, switch, and modification of originator *vs*. generic initiators after 1:1 propensity score matching.

Drug		Group	Sample size, n	Total person-years	Treatment discontinuation			Treatment switch			Treatment modification		
					Events, n	IR/1,000 person-years	HR (95%CI)	Events, n	IR/1,000 person-years	HR (95%CI)	Events, n	IR/1,000 person-years	HR (95%CI)
CCBs	Amlodipine	Originator	1,775	1,640	956	582.9	1.28 (1.17, 1.39)	16	582.9	1.85 (0.84, 4.08)	284	173.2	0.74 (0.63, 0.86)
		Generic	1,775	1,710	801	468.4	Reference	10	468.4	Reference	436	255	Reference
	Felodipine	Originator	2,157	1,906	1,329	697.3	1.23 (1.14, 1.32)	49	697.3	1.25 (0.84, 1.86)	274	143.8	0.96 (0.82, 1.13)
		Generic	2,157	2,171	1,184	545.4	Reference	48	545.4	Reference	349	160.8	Reference
	Nifedipine	Originator	936	840	551	656	1.04 (0.93, 1.15)	12	656	1.98 (0.76, 5.12)	120	142.9	0.94 (0.73, 1.20)
		Generic	936	786	546	694.7	Reference	6	694.7	Reference	133	169.2	Reference
ARBs	Irbesartan	Originator	645	581	332	571.4	1.20 (1.04, 1.39)	23	571.4	5.50 (2.07, 14.65)	104	179	1.00 (0.77, 1.31)
		Generic	645	621	304	489.5	Reference	5	489.5	Reference	124	199.7	Reference
	Losartan	Originator	1,612	1,389	974	701.2	1.31 (1.20, 1.43)	46	701.2	1.95 (1.22, 3.13)	208	149.7	0.76 (0.64, 0.90)
		Generic	1,612	1,468	794	540.9	Reference	27	540.9	Reference	315	214.6	Reference
	Valsartan	Originator	2,147	2,060	1,135	551	1.09 (1.01, 1.18)	39	551	0.97 (0.63, 1.50)	392	190.3	1.01 (0.88, 1.16)
		Generic	2,147	2,170	1,116	514.3	Reference	46	514.3	Reference	432	199.1	Reference
Beta-blocker	Metoprolol	Originator	1,263	1,073	865	806.2	1.29 (1.18, 1.40)	21	806.2	0.86 (0.49, 1.52)	150	139.8	1.28 (1.01, 1.60)
		Generic	1,263	1,122	743	662.2	Reference	31	662.2	Reference	142	126.6	Reference

Abbreviations: IR, incidence ratio; HR, hazard ratio; CI, confidence interval. CCB, calcium channel blocker; ARB, angiotensin receptor blocker

### Sensitivity Analysis

Results of subgroup analyses were similar to primary analysis (Supplementary Figure S1A and Supplementary Table S10–S12). In the subgroup analysis of age, no significant differences were observed in the hospitalization rates between originator and generic group for nifedipine initiators aged <65 years and aged ≥65 years (HR [95% CI]: 4.61[0.96, 22.20] and 3.18[0.89, 11.30], respectively), and felodipine initiators aged <65 years (HR [95% CI]: 1.03[0.34, 3.10]). A significantly higher hospitalization rate was found in the originator group for metoprolol initiators aged ≥65 years (HR [95% CI]: 4.62[1.34, 15.96]) (Supplementary Figure S1). For patients without treatment discontinuation within 180 days in the follow-up, no significant difference was observed in originator and generic initiators of nifedipine (HR [95% CI]: 1.89[0.78, 4.61]), and significantly higher hospitalization rates were founded in originator initiators of irbesartan and losartan (HR [95% CI]: 2.57[1.39, 4.74] and 3.85[1.30, 11.39], respectively) (Supplementary Table S10).

As induction time became shorter, higher estimated hazard ratios of hospitalization were observed between originator and generic initiators of irbesartan, losartan, and valsartan (Supplemenatary Table S11). Meanwhile, given different induction and latent time, significantly higher hypertension-related costs for originator initiators were found as in prior analysis (Supplementary Table S12).

## Discussion

Our findings indicated comparable or even better clinical effectiveness and lower hypertension-related medical costs in generic antihypertensive drug initiators compared with those in originator initiators. As the first study to compare the clinical outcomes and medical costs of originator and generic drugs in China, we provided critical evidence for generic substitution and clinical practice.

Consistent with most studies on generics and the pooled result of random controlled trials, our study found comparable clinical outcomes of generics and originator products for hospital visits (Desai et al., 2019; Gagne et al., 2014; Gagne et al., 2015). Noticeably, we found lower hospitalization rates for CVD in generic initiators for three CCB drugs out of the seven drug cohorts, which could be attributable to different levels of medication adherence in the two patient groups. In this study, generic initiators were less likely to discontinue their treatment compared with originator initiators. This finding echoed previous studies in which patients treated with generics experienced better clinical outcomes (Corrao et al., 2014; Gagne et al., 2014).

Besides, we found substantially lower medical costs in generic initiators, indicating the potential of generic substitution to save drug costs. Hypertension was the primary risk factor for cardiovascular disease, a leading cause of mortality in China (Lewington et al., 2016). As originator antihypertensive drugs implied a significant financial commitment, only 23% of hypertension patients in China regularly took originator antihypertensive drugs, and less than 16% had effective blood pressure control (Ho et al., 2009; Lu et al., 2017; Su et al., 2017). Besides, higher cost may negatively impact patient adherence to medicines and thus clinical outcomes (Sinnott et al., 2013; Mann et al., 2014; Simoens and Sinnaeve, 2014; Banerjee et al., 2016). Given the comparable clinical effectiveness of generics, patients and healthcare providers can be reassured to preferentially use generics to lower drug costs and improve medication adherence and ultimately blood pressure control rate. Therefore, we suggest Chinese regulators to promote generics use and establish relevant health policies of generic substitution (Shrank et al., 2010; Mishuk et al., 2020).

Our study had several strengths. Compared with prior observational studies, we balanced potential confounding through propensity score matching, which was used in only a few previous studies and made our results more robust and reliable (Leclerc et al., 2020). Besides, we required a 180-day antihypertensive drug-naive period before treatment initiation and considered the incubation and latent time; these designs further controlled for unmeasured confounding factors, such as hypertension history and the dose modification at the beginning of follow-up.

However, our study also had several limitations. First, we included drug use information and CCI score in the baseline period to balance baseline clinical characteristics of patients between originator and generic treatment groups. Nevertheless, blood pressure, body mass index, and other variables were missing from the data, making it difficult to fully capture the health status of individual patients. Second, we failed to obtain all patients' income information in the dataset. Previous studies demonstrated that high-income patients tended to use originator products and be hospitalized for mild symptoms (Zhao et al., 2019), probably leading to higher hospitalization rates (Vrijens et al., 2012). However, we included 18,118 patients with income information in the additional analysis and found a non-significant impact of income on initiating originator products or generics (Supplementary Table S13). Furthermore, given that whether the patients chose to be hospitalized could be influenced by the severity of diseases and income, we adopted hospitalization for MI, stroke, and CHF as the secondary outcome (Lin et al., 2018), and the results suggested our primary analysis result remained valid (Supplementary Figure S2). Third, we did not distinguish between generic products of the same INN from different manufactures; thus, further studies need to investigate the clinical effectiveness of individual generics from different manufacturers. Fourth, we only included patients treated with monotherapy, which comprised 81.5% of all patients treated for hypertension (Lu et al., 2017). Patients in our study were thus likely to represent a cohort with mild hypertension, as most severe hypertension patients need two or more antihypertensive drugs to effectively control blood pressure according to the guidelines (Wang et al., 2020). Fifth, similar to previous studies (Corrao et al., 2008; Corrao et al., 2014; Desai et al., 2019), the follow-up period of sample patients was relatively short due to complicated endpoints, including treatment discontinuation, modification, and switching. Sixth, immortal time bias might have been introduced by excluding patients who died or modified treatment within the 90-day incubation period. Last, our population was limited to residents of Yinzhou, which is a district in Ningbo, an economically developed coastal city of southeast China. Thus, our findings should be extrapolated with caution.

## Conclusion

We observed comparable or even better clinical outcomes and less medical cost associated with the antihypertensive generics compared with their originator counterparts. This could help increase health professional and patient confidence in the efficacy of generic medicines and promote the use of generics to manage hypertension.

## Data Availability

The original contributions presented in the study are included in the article/Supplementary Material, further inquiries can be directed to the corresponding author.

## Abbreviations

ARB, angiotensin receptor blockers; CVD, cardiovascular diseases; CCI, the Charlson comorbidity index; CCB, the calcium channel blockers; CHF, congestive heart failure; CHERRY, the Chinese Electronic Health Records Research in Yinzhou; CI, confidence interval; ED, emergency department; HR, hazard ratio; ICD-10, International Classification of Diseases Tenth Revision; INN, international nonproprietary name; IQR, interquartile range; MI, myocardial infarction; NMPA, the National Medical Products Administration; NSAID, nonsteroidal anti-inflammatory drug; SMD, standardized mean difference; UEBMI, the basic medical insurance for urban employees; URBMI, the basic medical insurance for urban residents.
