# Supplementary material for: Clinical Outcome and Medical Cost of Originator and Generic Antihypertensive Drugs: A Population-Based Study in Yinzhou, China
Source: Front Pharmacol. 2022 Feb 22;13:757398. doi: 10.3389/fphar.2022.757398 (PMC8920543; doi:10.3389/fphar.2022.757398)
Supplement: Supplementary file 1 [file DataSheet1.docx]

# Supplement Material

# List of content

**1.Antihypertensive drugs and baseline drug use information**

Table1………………………………………………………………………………..2

Table2………………………………………………………………………………..4

**2.Sample characteristics**

Table3………………………………………………………………………………..6

Table4………………………………………………………………………………..8

Table5………………………………………………………………………………10

Table6………………………………………………………………………………12

Table7………………………………………………………………………………14

Table8………………………………………………………………………………16

Table9………………………………………………………………………………18

**3.Sensitive analysis**

**Subgroup analysis**

Figure1……………………………………………………………………………...20

Table10……………………………………………………………………….……..21

**Different induction and latent time**

Table11……………………………………………………………………………..22

Table12……………………………………………………………………………..24

**Income analysis**

Table13……………………………………………………………………………..29

**Secondary outcome**

Figure2……………………………………………………………………………...30

## Antihypertensive drugs and Baseline drug use information

**Table1** Antihypertensive drugs information.

| **Category** | **Drug** | **ATC code** |
| --- | --- | --- |
| **CCBs** | nifedipine, amlodipine, lekadipine (lercanidipine), nimodipine, nicardipine, nigandipine (nitrendipine), nisoldipine, felodipine, benidipine, lacidipine, isradipine, diltiazem, verapamil | C05AE03，C07FBxx，C08CA01-C08CA09，C08CA13，C08CA15，C08CA55，C08DA01，C08DA51，C08DBxx，C08GAxx，C09BB02-C09BB07，C09BB10，C09BX01，C09BX03，C09DBxx，C09DX01，C09DX03，C09DX06，C09XA53 |
| **Beta-blockers** | atenolol, metoprolol, propranolol, carvedilol, bisoprolol | C07AA05，C07AB02，C07AB03，C07AB07，C07AB11，C07AG02，C07BA05，C07BB02，C07BB03，C07BB07，C07BB52，C07CBxx，C07DBxx，C07FB02，C07FB07，C07FB13，C07FX01，C07FX03-C07FX06，C09BX02 |
| **Diuretics** | furosemide, tolasemide (torasemide), hydrochlorothiazide, bendroflumethiazide, indapamide, chlorothiazide, amiloride, spironolactone, chlorthalidone (chlortalidone), methyclothiazide, trichlormethiazide, metolazone, indapamide, eplerenone, etacrynic acid, bumetanide | C03AA01，C03AA03，C03AA04，C03AA06，C03AA08，C03AB01，C03AB03，C03AB04，C03AB06，C03AB08，C03AH01，C03BA04，C03BA08，C03BA11，C03BB04，C03CA01，C03CA02，C03CA04，C03CBxx，C03CC01，C03DA01，C03DA04，C03DB01，C03EA01，C03EA02，C03EA06，C03EA12，C03EA13，C03EBxx |
| **ACEIs** | captopril, enalapril, benazepril, imidapril, lisinopril, perindopril, ramipril, trandolapril, lisinopril, cilazapril, fosinopril, moexipril, quinapril | C09AA01-C09AA10，C09AA13，C09AA16，C09BA01，C09BA09，C09BA13，C09BB02-C09BB07，C09BB10，C09BXxx，C09DA04，C09DX01，C09DX03，C09DX06，C09XA52，C09XA54，C10BX06，C10BX07，C10BX11-C10BX15 |
| **ARBs** | clorsartan (losartan), valsartan, irbesartan, candesartan, telmisartan, eprosartan, azilsartan, olmesartan | C09CA01-C09CA04，C09CA06-C09CA09，C09DA01-C09DA04，C09DA06-C09DA09，C09DBxx，C09DXxx，C10BX10 |
| **Centrally acting antihypertensive drug** | clonidine, methyldopa, rilmenidine, moxonidine | C02ABxx，C02AC01，C02AC05，C02AC06，C02LBxx，C02LCxx，N02CX02，S01EA04 |
| **Alpha-blockers** | terazosin, doxazosin, prazosin, trimazosin, urapidil | C02CA01，C02CA03，C02CA04，C02CA06，C02LExx，G04CA03 |
| **Compounds** | amlodipine and hydrochlorothiazide, nifedipine and hydrochlorothiazide, losartan and hydrochlorothiazide, valsartan and hydrochlorothiazide, reserpine(combinations) | C09BX03，C09DX01，C09DX03，C09DX06，C09XA54, C02AA52, C02AA52 |
| **Others** | reserpine | C02AA02 |
| **Traditional Chinese Medicine** | jiangyalinghao, zhenjujiangya, fufangluobuma, fufangluding, | NA |

Note: NA=not available.

**Table2** Drug use information in the baseline period.

| **Category** | **Drug name** | **ATC code** |
| --- | --- | --- |
| **Statins** | atorvastatin, fluvastatin, lovastatin, pravastatin, simvastatin, rosuvastatin, pitavastatin | C10AA05, C10AA04, C10AA02, C10AA03, C10AA01, C10AA07, C10AA08 |
| **Other lipid lowering drugs** | acipimox, probucol, fenofibrate, bezafibrate, gemfibrozil, olestyramine, colestipol, ezetimibe | C10AD06, C10AX02, C10AB05, C10AB02, C10AB04, C10AC01, C10AC02, C10AX09 |
| **Antiplatelets** | acetylsalicylic acid, clopidogrel, prasugrel, ticagrelor, abciximab, tirofiban, dipyridamole, cilostazol | A01AD05, B01AC06, N02BA01, B01AC04, B01AC22, B01AC24, B01AC13, B01AC17, B01AC07, B01AC23 |
| **Insulin preparations** | insulin (human), insulin (beef), insulin (pork), insulin lispro, insulin aspart, insulin glulisine, insulin glargine, insulin detemir, insulin degludec | A10AB01-A10AB06, A10AC01-A10AC04, A10AD01-A10AD05, A10AE01-A10AE06, A10AF01 |
| **Oral hypoglycemic agents** | metformin, gliquidone, glipizide, glimepiride, gliclazide, glibenclamide, repaglinide, nateglinide, mitiglinide, acarbose, voglibose, miglitol, rosiglitazone, pioglitazone, exenatide, liraglutide, sitagliptin, vildagliptin, saxagliptin, linagliptin, | A10BA02, A10BB08, A10BB07, A10BB12, A10BB09, A10BB01, A10BF01, A10BF03, A10BF02, A10BG02, A10BG03, A10BH01, A10BH04, A10BH02, A10BH03, A10BH05, A10BJ01, A10BJ02, A10BX02, A10BX03, A10BX08 |
| **Aspirin** | acetylsalicylic acid | A01AD05, B01AC06, N02BA01 |
| **Other NSAIDs** | sodium salicylate; salsalate; sulfasalazine; paracetamol; indometacin; sulindac; etodolac; tolmetin; diclofenac; ibuprofen; dexibuprofen; flurbiprofen; ketoprofen; dexketoprofen; fenoprofen; naproxen; piketoprofen; mefenamic acid; meclofenamic acid; piroxicam; lornoxicam; tenoxicam; nabumetone; | N02BA04, N02BA06, A07EC01, N02BE01, C01EB03, M01AB01, M02AA23, S01BC01, M01AB02, M01AB08, M01AB03, M02AA21, D11AX18, M01AB05, M02AA15, S01BC03, C01EB16, G02CC01, M01AE01, M01AE14, M02AA13, R02AX02, G02CC02, M01AE02, M02AA12, M01AE09, M02AA19, R02AX01, S01BC04, M01AE03, M01AE17, M02AA10, M02AA27, M02AA28, M01AE04, M01AG01, M01AG04, M02AA18, M01AC01, M02AA07, S01BC06, M01AC05, M01AC02, M01AX01, |
| **Nitrates** | glyceryl trinitrate, isosorbide dinitrate, isosorbide mononitrate, ranolazine | C01DA02, C05AE01, C01DA08, C05AE02, C01DA14, C01EB18 |
| **Anticoagulants** | warfarin, argatroban, bivalirudin, fondaparinux, heparin, organo-heparinoid, iodoheparinate, nadroparin, enoxaparin, dalteparin | B01AA031, B01AE03, B01AE06, B01AX05, B01AB01, C05BA01, C05BA03, S01XA09, S01XA14, B01AB06, B01AB05, B01AB04 |
| **Digoxin** | digoxin, acetyldigoxin, metildigoxin, lanatoside C | C01AA05, C01AA02, C01AA08, C01AA06 |
| **Anti-arrhythmics** | adenosine, amiodarone, quinidine, hydroquinidine, lidocaine；mexiletine, propafenone | C01EB10, C01BD01, C01BA01, C01BA13, C01BB01, C05AD01, D04AB01, N01BB02, R02AD02, S01HA07, S02DA01, C01BB02, C01BC03 |
| **Coxibs** | celecoxib, rofecoxib, valdecoxib, parecoxib, etoricoxib, lumiracoxib, polmacoxib | M01AHxx |

Abbreviations: NSAIDs, nonsteroidal anti-inflammatory drugs.

## Sample characteristics

**Table3** Characteristics of patients included in the evaluation of comparative outcomes for **amlodipine** between originator and generic initiators.

| **Sample Characteristics** | | **Before propensity score Matching** | | | **After propensity score Matching** | | |
| --- | --- | --- | --- | --- | --- | --- | --- |
|  |  | **Generic** | **Originator** | **SMD** | **Generic** | **Originator** | **SMD** |
| **N** | | 3362 | 2026 |  | 1775 | 1775 |  |
| **Age, mean(sd)** | | 61.1(11.7) | 62.8(12.7) | 0.13882 | 61.9(11.7) | 61.3(12.1) | 0.05317 |
| **Gender, n(%)** | **Male** | 1649(49.0) | 994(49.1) | 0.00028 | 877(49.4) | 885(49.9) | 0.00901 |
|  | **Female** | 1713(51.0) | 1032(50.9) |  | 898(50.6) | 890(50.1) |  |
| **Insurance type, n(%)** | **URBMI/UEBMI** | 1922(57.2) | 1730(85.4) | 0.65850 | 1474(83.0) | 1482(83.5) | 0.05566 |
|  | **NCMS** | 1401(41.7) | 282(13.9) |  | 294(16.6) | 279(15.7) |  |
|  | **Without insurance** | 39(1.2) | 14(0.7) |  | 7(0.4) | 14(0.8) |  |
| **Outpatient visits, mean(sd)** | | 4.1(5.6) | 3.0(4.76) | 0.21459 | 3.0(4.5) | 3.0(4.9) | 0.00824 |
| **ED visits, n(%)** | **≥1** | 110(3.3) | 49(2.4) | 0.05134 | 48(2.7) | 45(2.5) | 0.01058 |
| **Hospitalizations, n(%)** | **≥1** | 37(1.1) | 41(2) | 0.07450 | 23(1.3) | 12(0.7) | 0.06275 |
| **Charlson index score, mean(sd)** | | 0.3(0.7) | 0.3(0.8) | 0.02186 | 0.3(0.8) | 0.3(0.7) | 0.04345 |
| **Charlson index score, n(%)** | **0** | 2679(79.7) | 1615(79.7) | 0.03372 | 1452(81.8) | 1482(83.5) | 0.05005 |
|  | **1** | 500(14.9) | 287(14.2) |  | 228(12.8) | 214(12.1) |  |
|  | **≥2** | 183(5.4) | 124(6.1) |  | 95(5.4) | 79(4.5) |  |
| **Index year, n(%)** | **2012** | 630(18.7) | 298(14.7) | 0.30290 | 248(14.0) | 264(14.9) | 0.04887 |
|  | **2013** | 446(13.3) | 272(13.4) |  | 221(12.5) | 201(11.3) |  |
|  | **2014** | 438(13.0) | 228(11.3) |  | 211(11.9) | 205(11.5) |  |
|  | **2015** | 366(10.9) | 185(9.1) |  | 165(9.3) | 162(9.1) |  |
|  | **2016** | 593(17.6) | 242(11.9) |  | 244(13.7) | 237(13.4) |  |
|  | **2017** | 720(21.4) | 660(32.6) |  | 575(32.4) | 586(33) |  |
|  | **2018** | 169(5.0) | 141(7) |  | 111(6.3) | 120(6.8) |  |
| **Statins, n(%)** | | 148(4.4) | 119(5.9) | 0.06669 | 86(4.8) | 63(3.5) | 0.06465 |
| **Oral hypoglycemic agents, n(%)** | | 211(6.3) | 92(4.5) | 0.07677 | 83(4.7) | 82(4.6) | 0.00268 |
| **Insulin preparations, n(%)** | | 13(0.4) | 17(0.8) | 0.05799 | 9(0.5) | 5(0.3) | 0.03596 |
| **Anticoagulants, n(%)** | | 2(0.1) | 4(0.2) | 0.03852 | 2(0.1) | 1(0.1) | 0.01939 |
| **Antiplatelets, n(%)** | | 8(0.2) | 11(0.5) | 0.04892 | 5(0.3) | 3(0.2) | 0.02376 |
| **Nitrates, n(%)** | | 15(0.4) | 17(0.8) | 0.04919 | 8(0.5) | 3(0.2) | 0.05070 |
| **Other lipid-lowering agents, n(%)** | | 19(0.6) | 9(0.4) | 0.01706 | 7(0.4) | 9(0.5) | 0.01682 |
| **Aspirin, n(%)** | | 101(3.0) | 54(2.7) | 0.02042 | 43(2.4) | 40(2.3) | 0.01119 |
| **Other NSAIDs, n(%)** | | 219(6.5) | 118(5.8) | 0.02867 | 101(5.7) | 87(4.9) | 0.03522 |
| **Coxibs, n(%)** | | 14(0.4) | 9(0.4) | 0.00425 | 7(0.4) | 8(0.5) | 0.00869 |
| **Digoxin, n(%)** | | 3(0.1) | 1(0.0) | 0.01515 | 2(0.1) | 1(0.1) | 0.01939 |

Abbreviations: propensity score, propensity score; SMD, standard mean difference; URBMI, Urban Residents Basic Health Insurance, UEBMI, Urban Employee Basic Health Insurance; NRCMS, New Rural Cooperative Medical Scheme; ED, emergency department; NSAIDs, nonsteroidal anti-inflammatory drugs.

**Table4** Characteristics of patients included in the evaluation of comparative outcomes for **felodipine** between originator and generic initiators.

| **Sample Characteristics** | | **Before propensity score Matching** | | | **After propensity score Matching** | | |
| --- | --- | --- | --- | --- | --- | --- | --- |
|  |  | **Generic** | **Originator** | **SMD** | **Generic** | **Originator** | **SMD** |
| **N** | | 4248 | 2698 |  | 2157 | 2157 |  |
| **Age, mean(sd)** | | 61.8(11.4) | 62.2(11.9) | 0.02914 | 61.5(11.4) | 60.8(11.6) | 0.06091 |
| **Gender, n(%)** | **Male** | 2051(48.3) | 1388(51.4) | 0.06331 | 1072(49.7) | 1077(49.9) | 0.00464 |
|  | **Female** | 2197(51.7) | 1310(48.6) |  | 1085(50.3) | 1080(50.1) |  |
| **Insurance type, n(%)** | **URBMI/UEBMI** | 1885(44.4) | 2156(79.9) | 0.78971 | 1616(74.9) | 1628(75.5) | 0.01594 |
|  | **NCMS** | 2334(54.9) | 528(19.6) |  | 525(24.3) | 515(23.9) |  |
|  | **Without insurance** | 29(0.7) | 14(0.5) |  | 16(0.7) | 14(0.6) |  |
| **Outpatient visits, mean(sd)** | | 4.8(6.3) | 3.5(5.3) | 0.21013 | 3.7(5.1) | 3.8(5.4) | 0.02311 |
| **ED visits, n(%)** | **≥1** | 111(2.6) | 71(97.4) | 0.00116 | 53(2.5) | 49(2.3) | 0.01221 |
| **Hospitalizations, n(%)** | **≥1** | 24(0.6) | 76(2.8) | 0.17533 | 21(1.0) | 9(0.4) | 0.06698 |
| **Charlson index score, mean(sd)** | | 0.3(0.7) | 0.4(0.9) | 0.07043 | 0.3(0.8) | 0.3(0.7) | 0.03949 |
| **Charlson index score, n(%)** | **0** | 3376(79.5) | 2134(79.1) | 0.07659 | 1735(80.4) | 1746(80.9) | 0.04624 |
|  | **1** | 642(15.1) | 372(13.8) |  | 294(13.6) | 305(14.1) |  |
|  | **≥2** | 230(5.4) | 192(7.1) |  | 128(5.9) | 106(4.9) |  |
| **Index year, n(%)** | **2012** | 1466(34.5) | 543(20.1) | 0.45323 | 505(23.4) | 516(23.9) | 0.17767 |
|  | **2013** | 688(16.2) | 406(15) |  | 316(14.6) | 365(16.9) |  |
|  | **2014** | 459(10.8) | 293(10.9) |  | 252(11.7) | 266(12.3) |  |
|  | **2015** | 415(9.8) | 265(9.8) |  | 207(9.6) | 235(10.9) |  |
|  | **2016** | 677(15.9) | 426(15.8) |  | 408(18.9) | 407(18.9) |  |
|  | **2017** | 437(10.3) | 638(23.6) |  | 383(17.8) | 257(11.9) |  |
|  | **2018** | 106(2.5) | 127(4.7) |  | 86(4) | 111(5.1) |  |
| **Statins, n(%)** | | 174(4.1) | 114(4.2) | 0.00648 | 85(3.9) | 77(3.6) | 0.01951 |
| **Oral hypoglycemic agents, n(%)** | | 250(5.9) | 167(6.2) | 0.01279 | 128(5.9) | 116(5.4) | 0.02409 |
| **Insulin preparations, n(%)** | | 22(0.5) | 30(1.1) | 0.06611 | 17(0.8) | 6(0.3) | 0.07007 |
| **Anticoagulants, n(%)** | | 1(0.0) | 10(0.4) | 0.07832 | 1(0.0) | 0(0) | 0.03046 |
| **Antiplatelets, n(%)** | | 18(0.4) | 16(0.6) | 0.02381 | 7(0.3) | 11(0.5) | 0.02877 |
| **Nitrates, n(%)** | | 28(0.7) | 8(0.3) | 0.09512 | 17(0.8) | 14(0.6) | 0.01647 |
| **Other lipid-lowering agents, n(%)** | | 46(1.1) | 8(0.3) | 0.09512 | 4(0.2) | 6(0.3) | 0.01928 |
| **Aspirin, n(%)** | | 83(2.0) | 91(3.4) | 0.08822 | 60(2.8) | 44(2.0) | 0.04837 |
| **Other NSAIDs, n(%)** | | 288(6.8) | 182(6.7) | 0.00135 | 142(6.6) | 141(6.5) | 0.00187 |
| **Coxibs, n(%)** | | 8(0.2) | 11(0.4) | 0.04026 | 6(0.3) | 7(0.3) | 0.00846 |
| **Digoxin, n(%)** | | 5(0.1) | 3(0.1) | 0.00193 | 1(0.0) | 1(0.0) | 0.00000 |

Abbreviations: propensity score, propensity score; SMD, standard mean difference; URBMI, Urban Residents Basic Health Insurance, UEBMI, Urban Employee Basic Health Insurance; NRCMS, New Rural Cooperative Medical Scheme; ED, emergency department; NSAIDs, nonsteroidal anti-inflammatory drugs.

**Table5** Characteristics of patients included in the evaluation of comparative outcomes for **nifedipine** between originator and generic initiators.

| **Sample Characteristics** | | **Before propensity score Matching** | | | **After propensity score Matching** | | |
| --- | --- | --- | --- | --- | --- | --- | --- |
|  |  | **Generic** | **Originator** | **SMD** | **Generic** | **Originator** | **SMD** |
| **N** | | 1953 | 1355 |  | 936 | 936 |  |
| **Age, mean(sd)** | | 64.5(11.9) | 63.3(13.3) | 0.09744 | 62.9(12.4) | 63.6(13.6) | 0.05201 |
| **Gender, n(%)** | **Male** | 842(43.1) | 677(50.0) | 0.13765 | 452(48.3) | 437(46.7) | 0.03210 |
|  | **Female** | 1111(56.9) | 678(50.0) |  | 484(51.7) | 499(53.3) |  |
| **Insurance type, n(%)** | **URBMI/UEBMI** | 806(41.3) | 1069(78.9) | 0.83939 | 651(69.6) | 659(70.4) | 0.05298 |
|  | **NCMS** | 1133(58.0) | 275(20.3) |  | 279(29.8) | 267(28.5) |  |
|  | **Without insurance** | 14(0.7) | 11(0.8) |  | 6(0.6) | 10(1.1) |  |
| **Outpatient visits, mean(sd)** | | 4.4(5.9) | 3.2(5.1) | 0.22479 | 3.7(4.8) | 3.7(5.5) | 0.00374 |
| **ED visits, n(%)** | **≥1** | 51(2.6) | 56(4.1) | 0.08436 | 35(3.7) | 27(2.9) | 0.04778 |
| **Hospitalizations, n(%)** | **≥1** | 14(0.7) | 52(3.8) | 0.21035 | 11(1.2) | 4(0.4) | 0.08396 |
| **Charlson index score, mean(sd)** | | 0.3(0.7) | 0.3(0.8) | 0.01659 | 0.3(0.7) | 0.3(0.7) | 0.00147 |
| **Charlson index score, n(%)** | **0** | 1525(78.1) | 1049(77.4) | 0.02464 | 728(77.8) | 728(77.8) | 0.00000 |
|  | **1** | 318(16.3) | 222(16.4) |  | 158(16.9) | 158(16.9) |  |
|  | **≥2** | 110(5.6) | 84(6.2) |  | 50(5.3) | 50(5.3) |  |
| **Index year, n(%)** | **2012** | 750(38.4) | 205(15.1) | 0.74495 | 171(18.3) | 199(21.3) | 0.2618 |
|  | **2013** | 316(16.2) | 143(10.6) |  | 133(14.2) | 134(14.3) |  |
|  | **2014** | 183(9.4) | 164(12.1) |  | 113(12.1) | 137(14.6) |  |
|  | **2015** | 127(6.5) | 147(10.8) |  | 95(10.1) | 99(10.6) |  |
|  | **2016** | 341(17.5) | 218(16.1) |  | 208(22.2) | 193(20.6) |  |
|  | **2017** | 168(8.6) | 376(27.7) |  | 157(16.8) | 86(9.2) |  |
|  | **2018** | 68(3.5) | 102(7.5) |  | 59(6.3) | 88(9.4) |  |
| **Statins, n(%)** | | 100(5.1) | 86(6.3) | 0.05278 | 49(5.2) | 50(5.3) | 0.00477 |
| **Oral hypoglycemic agents, n(%)** | | 137(7.0) | 74(5.5) | 0.06427 | 59(6.3) | 60(6.4) | 0.00438 |
| **Insulin preparations, n(%)** | | 12(0.6) | 17(1.3) | 0.06657 | 7(0.7) | 6(0.6) | 0.01287 |
| **Anticoagulants, n(%)** | | 1(0.1) | 18(1.3) | 0.13731 | 1(0.1) | 0(0) | 0.04625 |
| **Antiplatelets, n(%)** | | 3(0.2) | 18(1.3) | 0.13731 | 2(0.2) | 1(0.1) | 0.02671 |
| **Nitrates, n(%)** | | 17(0.9) | 28(2.1) | 0.09955 | 11(1.2) | 11(1.2) | 0.00000 |
| **Other lipid-lowering agents, n(%)** | | 12(0.6) | 4(0.3) | 0.04746 | 3(0.3) | 2(0.2) | 0.02070 |
| **Aspirin, n(%)** | | 51(2.6) | 40(3.0) | 0.02072 | 26(2.8) | 26(2.8) | 0.00000 |
| **Other NSAIDs, n(%)** | | 148(7.6) | 75(5.5) | 0.08261 | 65(6.9) | 62(6.6) | 0.01275 |
| **Coxibs, n(%)** | | 2(0.1) | 7(0.5) | 0.07462 | 2(0.2) | 3(0.3) | 0.02070 |
| **Digoxin, n(%)** | | 5(0.3) | 0(0) | 0.07165 | 1(0.1) | 0(0) | 0.04625 |

Abbreviations: propensity score, propensity score; SMD, standard mean difference; URBMI, Urban Residents Basic Health Insurance, UEBMI, Urban Employee Basic Health Insurance; NRCMS, New Rural Cooperative Medical Scheme; ED, emergency department; NSAIDs, nonsteroidal anti-inflammatory drugs.

**Table6** Characteristics of patients included in the evaluation of comparative outcomes for **irbesartan** between originator and generic initiators.

| **Sample Characteristics** | | **Before propensity score Matching** | | | **After propensity score Matching** | | |
| --- | --- | --- | --- | --- | --- | --- | --- |
|  |  | **Generic** | **Originator** | **SMD** | **Generic** | **Originator** | **SMD** |
| **N** | | 8574 | 646 |  | 645 | 645 |  |
| **Age, mean(sd)** | | 60.2(11.5) | 61.9(12.1) | 0.14199 | 61.7(11.7) | 61.9(12.1) | 0.01238 |
| **Gender, n(%)** | **Male** | 4039(47.1) | 331(51.2) | 0.08270 | 344(53.3) | 330(51.2) | 0.04347 |
|  | **Female** | 4535(52.9) | 315(48.8) |  | 301(46.7) | 315(48.8) |  |
| **Insurance type, n(%)** | **URBMI/UEBMI** | 5303(61.8) | 596(92.3) | 0.78440 | 604(93.6) | 595(92.2) | 0.05493 |
|  | **NCMS** | 3213(37.5) | 46(7.1) |  | 38(5.9) | 46(7.1) |  |
|  | **Without insurance** | 58(0.7) | 4(0.6) |  | 3(0.5) | 4(0.6) |  |
| **Outpatient visits, mean(sd)** | | 4.1(5.7) | 3.2(5.0) | 0.16884 | 2.8(4.1) | 3.2(5.0) | 0.09100 |
| **ED visits, n(%)** | **≥1** | 280(3.3) | 10(1.5) | 0.11225 | 13(2.0) | 10(1.6) | 0.03515 |
| **Hospitalizations, n(%)** | **≥1** | 82(1.0) | 33(5.1) | 0.24393 | 28(4.3) | 32(5.0) | 0.02945 |
| **Charlson index score, mean(sd)** | | 0.3(0.8) | 0.5(1.0) | 0.14694 | 0.4(1.0) | 0.5(1.0) | 0.04518 |
| **Charlson index score, n(%)** | **0** | 6699(78.1) | 475(73.5) | 0.16556 | 489(75.8) | 475(73.6) | 0.10373 |
|  | **1** | 1358(15.8) | 103(15.9) |  | 108(16.7) | 103(16) |  |
|  | **≥2** | 517(6.0) | 68(10.5) |  | 48(7.4) | 67(10.4) |  |
| **Index year, n(%)** | **2012** | 1675(19.5) | 69(10.7) | 0.56791 | 74(11.5) | 69(10.7) | 0.07920 |
|  | **2013** | 1393(16.2) | 60(9.3) |  | 57(8.8) | 59(9.1) |  |
|  | **2014** | 1033(12) | 40(6.2) |  | 42(6.5) | 40(6.2) |  |
|  | **2015** | 862(10.1) | 61(9.4) |  | 65(10.1) | 61(9.5) |  |
|  | **2016** | 1546(18) | 101(15.6) |  | 90(14) | 101(15.7) |  |
|  | **2017** | 1682(19.6) | 258(39.9) |  | 269(41.7) | 258(40.0) |  |
|  | **2018** | 383(4.5) | 57(8.8) |  | 48(7.4) | 57(8.8) |  |
| **Statins, n(%)** | | 405(4.7) | 42(6.5) | 0.07731 | 35(5.4) | 41(6.4) | 0.03951 |
| **Oral hypoglycemic agents, n(%)** | | 508(5.9) | 51(7.9) | 0.07773 | 46(7.1) | 50(7.8) | 0.02363 |
| **Insulin preparations, n(%)** | | 60(0.7) | 16(2.5) | 0.14249 | 18(2.8) | 15(2.3) | 0.02946 |
| **Anticoagulants, n(%)** | | 5(0.1) | 0(0) | 0.03416 | 0(0) | 0(0) | / |
| **Antiplatelets, n(%)** | | 20(0.2) | 4(0.6) | 0.05927 | 6(0.9) | 4(0.6) | 0.03536 |
| **Nitrates, n(%)** | | 72(0.8) | 5(0.8) | 0.00735 | 2(0.3) | 5(0.8) | 0.06334 |
| **Other lipid-lowering agents, n(%)** | | 57(0.7) | 6(0.9) | 0.02970 | 9(1.4) | 6(0.9) | 0.04340 |
| **Aspirin, n(%)** | | 188(2.2) | 29(4.5) | 0.12806 | 18(2.8) | 29(4.5) | 0.09111 |
| **Other NSAIDs, n(%)** | | 560(6.5) | 13(2.0) | 0.22487 | 18(2.8) | 13(2.0) | 0.05063 |
| **Coxibs, n(%)** | | 38(0.4) | 4(0.6) | 0.02421 | 7(1.1) | 4(0.6) | 0.05060 |

Abbreviations: propensity score, propensity score; SMD, standard mean difference; URBMI, Urban Residents Basic Health Insurance, UEBMI, Urban Employee Basic Health Insurance; NRCMS, New Rural Cooperative Medical Scheme; ED, emergency department; NSAIDs, nonsteroidal anti-inflammatory drugs.

**Table7** Characteristics of patients included in the evaluation of comparative outcomes for **losartan** between originator and generic initiators.

| **Sample Characteristics** | | **Before propensity score Matching** | | | **After propensity score Matching** | | |
| --- | --- | --- | --- | --- | --- | --- | --- |
|  |  | **Generic** | **Originator** | **SMD** | **Generic** | **Originator** | **SMD** |
| **N** | | 3630 | 1812 |  | 1612 | 1612 |  |
| **Age, mean(sd)** | | 59.2(11.8) | 59.7(12.7) | 0.04506 | 59.7(11.9) | 59.3(12.6) | 0.02799 |
| **Gender, n(%)** | **Male** | 1889(52.0) | 979(54.0) | 0.03988 | 877(54.4) | 867(53.8) | 0.01245 |
|  | **Female** | 1741(48.0) | 833(46.0) |  | 735(45.6) | 745(46.2) |  |
| **Insurance type, n(%)** | **URBMI/UEBMI** | 1931(53.2) | 1528(84.3) | 0.71313 | 1332(82.6) | 1337(82.9) | 0.01500 |
|  | **NCMS** | 1680(46.3) | 281(15.5) |  | 276(17.1) | 272(16.9) |  |
|  | **Without insurance** | 19(0.5) | 3(0.2) |  | 4(0.2) | 3(0.2) |  |
| **Outpatient visits, mean(sd)** | | 5.4(7.1) | 4.1(6.0) | 0.19152 | 3.8(5.3) | 3.9(5.9) | 0.00453 |
| **ED visits, n(%)** | **≥1** | 131(3.6) | 47(2.6) | 0.05858 | 37(2.3) | 41(2.5) | 0.01615 |
| **Hospitalizations, n(%)** | **≥1** | 37(1.0) | 69(3.8) | 0.18246 | 28(1.7) | 15(0.9) | 0.07034 |
| **Charlson index score, mean(sd)** | | 0.4(0.8) | 0.4(0.97) | 0.06255 | 0.4(0.8) | 0.3(0.8) | 0.06522 |
| **Charlson index score, n(%)** | **0** | 2711(74.7) | 1364(75.3) | 0.10713 | 1256(77.9) | 1297(80.5) | 0.06522 |
|  | **1** | 677(18.7) | 286(15.8) |  | 249(15.4) | 226(14.0) |  |
|  | **≥2** | 242(6.7) | 162(8.9) |  | 107(6.6) | 89(5.5) |  |
| **Index year, n(%)** | **2012** | 721(19.9) | 309(17.1) | 0.19651 | 253(15.7) | 262(16.3) | 0.05372 |
|  | **2013** | 508(14) | 228(12.6) |  | 191(11.8) | 195(12.1) |  |
|  | **2014** | 407(11.2) | 176(9.7) |  | 139(8.6) | 157(9.7) |  |
|  | **2015** | 386(10.6) | 178(9.8) |  | 154(9.6) | 161(10) |  |
|  | **2016** | 659(18.2) | 288(15.9) |  | 280(17.4) | 268(16.6) |  |
|  | **2017** | 757(20.9) | 487(26.9) |  | 471(29.2) | 448(27.8) |  |
|  | **2018** | 192(5.3) | 146(8.1) |  | 124(7.7) | 121(7.5) |  |
| **Statins, n(%)** | | 219(6) | 117(6.5) | 0.01752 | 95(5.9) | 78(4.8) | 0.04681 |
| **Oral hypoglycemic agents, n(%)** | | 221(6.1) | 162(8.9) | 0.10835 | 101(6.3) | 92(5.7) | 0.02354 |
| **Insulin preparations, n(%)** | | 30(0.8) | 36(2.0) | 0.09865 | 21(1.3) | 12(0.7) | 0.05549 |
| **Anticoagulants, n(%)** | | 3(0.1) | 6(0.3) | 0.05471 | 3(0.2) | 1(0.1) | 0.03525 |
| **Antiplatelets, n(%)** | | 11(0.3) | 33(1.8) | 0.14851 | 9(0.6) | 6(0.4) | 0.02735 |
| **Nitrates, n(%)** | | 29(0.8) | 22(1.2) | 0.04161 | 10(0.6) | 12(0.7) | 0.01507 |
| **Other lipid-lowering agents, n(%)** | | 21(0.6) | 13(0.7) | 0.01732 | 3(0.2) | 8(0.5) | 0.05321 |
| **Aspirin, n(%)** | | 72(2.0) | 72(4.0) | 0.11727 | 45(2.8) | 19(1.2) | 0.11582 |
| **Other NSAIDs, n(%)** | | 257(7.1) | 98(5.4) | 0.06912 | 86(5.3) | 85(5.3) | 0.00277 |
| **Coxibs, n(%)** | | 18(0.5) | 15(0.8) | 0.04095 | 8(0.5) | 4(0.2) | 0.04076 |
| **Digoxin, n(%)** | | 2(0.1) | 1(0.1) | 0.00004 | 1(0.1) | 1(0.1) | <0.00001 |

Abbreviations: propensity score, propensity score; SMD, standard mean difference; URBMI, Urban Residents Basic Health Insurance, UEBMI, Urban Employee Basic Health Insurance; NRCMS, New Rural Cooperative Medical Scheme; ED, emergency department; NSAIDs, nonsteroidal anti-inflammatory drugs.

**Table8** Characteristics of patients included in the evaluation of comparative outcomes for **valsartan** between originator and generic initiators.

| **Sample Characteristics** | | **Before propensity score Matching** | | | **After propensity score Matching** | | |
| --- | --- | --- | --- | --- | --- | --- | --- |
|  |  | **Generic** | **Originator** | **SMD** | **Generic** | **Originator** | **SMD** |
| **N** | | 5998 | 2271 |  | 2147 | 2147 |  |
| **Age, mean(sd)** | | 59.6(11.5) | 59.4(12.5) | 0.01016 | 59(11.6) | 59.1(12.4) | 0.01112 |
| **Gender, n(%)** | **Male** | 2928(48.8) | 1244(54.8) | 0.11952 | 1177(54.8) | 1170(54.5) | 0.00655 |
|  | **Female** | 3070(51.2) | 1027(45.2) |  | 970(45.2) | 977(45.5) |  |
| **Insurance type, n(%)** | **URBMI/UEBMI** | 3311(55.2) | 1924(84.7) | 0.68171 | 1803(84.0) | 1803(84.0) | 0.01458 |
|  | **NCMS** | 2646(44.1) | 337(14.8) |  | 336(15.6) | 334(15.6) |  |
|  | **Without insurance** | 41(0.7) | 10(0.4) |  | 8(0.4) | 10(0.5) |  |
| **Outpatient visits, mean(sd)** | | 4.9(6.6) | 3.3(5.2) | 0.27073 | 3.5(5.2) | 3.2(5.2) | 0.04999 |
| **ED visits, n(%)** | **≥1** | 139(2.3) | 51(2.2) | 0.00480 | 42(2.0) | 42(2.0) | 0.00000 |
| **Hospitalizations, n(%)** | **≥1** | 32(0.5) | 52(2.3) | 0.14928 | 24(1.1) | 14(0.7) | 0.04975 |
| **Charlson index score, mean(sd)** | | 0.3(0.8) | 0.3(0.9) | 0.00332 | 0.3(0.8) | 0.3(0.7) | 0.04460 |
| **Charlson index score, n(%)** | **0** | 4642(77.4) | 1819(80.1) | 0.09806 | 1754(81.7) | 1781(83.0) | 0.03307 |
|  | **1** | 1006(16.8) | 303(13.3) |  | 277(12.9) | 259(12.1) |  |
|  | **≥2** | 350(5.8) | 149(6.6) |  | 116(5.4) | 107(5.0) |  |
| **Index year, n(%)** | **2012** | 1138(19.0) | 349(15.4) | 0.43927 | 331(15.4) | 331(15.4) | 0.03243 |
|  | **2013** | 899(15.0) | 298(13.1) |  | 279(13.0) | 288(13.4) |  |
|  | **2014** | 866(14.4) | 243(10.7) |  | 214(10.0) | 231(10.8) |  |
|  | **2015** | 721(12.0) | 211(9.3) |  | 206(9.6) | 201(9.4) |  |
|  | **2016** | 1127(18.8) | 262(11.5) |  | 255(11.9) | 252(11.7) |  |
|  | **2017** | 1026(17.1) | 749(33) |  | 716(33.3) | 705(32.8) |  |
|  | **2018** | 221(3.7) | 159(7) |  | 146(6.8) | 139(6.5) |  |
| **Statins, n(%)** | | 271(4.5) | 110(4.8) | 0.01541 | 90(4.2) | 86(4.0) | 0.00940 |
| **Oral hypoglycemic agents, n(%)** | | 345(5.8) | 117(5.2) | 0.02643 | 96(4.5) | 87(4.1) | 0.02075 |
| **Insulin preparations, n(%)** | | 24(0.4) | 33(1.5) | 0.11006 | 17(0.8) | 7(0.3) | 0.06251 |
| **Anticoagulants, n(%)** | | 0(0) | 5(0.2) | 0.06643 | 0(0) | 4(0.2) | 0.06110 |
| **Antiplatelets, n(%)** | | 10(0.2) | 23(1.0) | 0.11067 | 9(0.4) | 3(0.1) | 0.05296 |
| **Nitrates, n(%)** | | 39(0.7) | 17(0.7) | 0.01180 | 15(0.7) | 10(0.5) | 0.03061 |
| **Other lipid-lowering agents, n(%)** | | 40(0.7) | 15(0.7) | 0.00079 | 12(0.6) | 11(0.5) | 0.00638 |
| **Aspirin, n(%)** | | 115(1.9) | 59(2.6) | 0.04583 | 45(2.1) | 44(2.0) | 0.00327 |
| **Other NSAIDs, n(%)** | | 359(6.0) | 129(5.7) | 0.01301 | 114(5.3) | 107(5) | 0.01476 |
| **Coxibs, n(%)** | | 18(0.3) | 14(0.6) | 0.04685 | 13(0.6) | 10(0.5) | 0.01914 |
| **Digoxin, n(%)** | | 5(0.1) | 4(0.2) | 0.02577 | 2(0.1) | 1(0.0) | 0.01763 |

Abbreviations: propensity score, propensity score; SMD, standard mean difference; URBMI, Urban Residents Basic Health Insurance, UEBMI, Urban Employee Basic Health Insurance; NRCMS, New Rural Cooperative Medical Scheme; ED, emergency department; NSAIDs, nonsteroidal anti-inflammatory drugs.

**Table9** Characteristics of patients included in the evaluation of comparative outcomes for **metoprolol** between originator and generic initiators.

| **Sample Characteristics** | | **Before propensity score Matching** | | | **After propensity score Matching** | | |
| --- | --- | --- | --- | --- | --- | --- | --- |
|  |  | **Generic** | **Originator** | **SMD** | **Generic** | **Originator** | **SMD** |
| **N** | | 2667 | 2097 |  | 1263 | 1263 |  |
| **Age, mean(sd)** | | 62.1(11.7) | 63.6(12.4) | 0.12099 | 61.7(12.0) | 61.2(12.3) | 0.04294 |
| **Gender, n(%)** | **Male** | 1196(44.8) | 915(43.6) | 0.02438 | 556(44.0) | 613(48.5) | 0.09061 |
|  | **Female** | 1471(55.2) | 1182(56.4) |  | 707(56.0) | 650(51.5) |  |
| **Insurance type, n(%)** | **URBMI/UEBMI** | 1045(39.2) | 1568(74.8) | 0.78615 | 822(65.1) | 806(63.8) | 0.03358 |
|  | **NCMS** | 1615(60.6) | 512(24.4) |  | 436(34.5) | 450(35.6) |  |
|  | **Without insurance** | 7(0.3) | 17(0.8) |  | 5(0.4) | 7(0.6) |  |
| **Outpatient visits, mean(sd)** | | 6.8(7.1) | 5.5(7.7) | 0.17133 | 5.5(6.5) | 5.7(8.0) | 0.02659 |
| **ED visits, n(%)** | **≥1** | 87(3.3) | 82(3.9) | 0.03487 | 40(3.2) | 40(3.2) | 0.00000 |
| **Hospitalizations, n(%)** | **≥1** | 21(0.8) | 77(3.7) | 0.19631 | 20(1.6) | 13(1.0) | 0.04882 |
| **Charlson index score, mean(sd)** | | 04(0.8) | 0.5(1.0) | 0.15379 | 0.4(0.9) | 0.4(0.8) | 0.01413 |
| **Charlson index score, n(%)** | **0** | 1976(74.1) | 1453(69.3) | 0.14773 | 941(74.5) | 948(75.1) | 0.01840 |
|  | **1** | 515(19.3) | 423(20.2) |  | 222(17.6) | 221(17.5) |  |
|  | **≥2** | 176(6.6) | 221(10.5) |  | 100(7.9) | 94(7.4) |  |
| **Index year, n(%)** | **2012** | 1201(45.0) | 369(17.6) | 0.98823 | 334(26.4) | 348(27.6) | 0.24881 |
|  | **2013** | 518(19.4) | 207(9.9) |  | 195(15.4) | 194(15.4) |  |
|  | **2014** | 377(14.1) | 223(10.6) |  | 209(16.5) | 199(15.8) |  |
|  | **2015** | 214(8.0) | 283(13.5) |  | 188(14.9) | 192(15.2) |  |
|  | **2016** | 275(10.3) | 491(23.4) |  | 255(20.2) | 306(24.2) |  |
|  | **2017** | 69(2.6) | 407(19.4) |  | 69(5.5) | 23(1.8) |  |
|  | **2018** | 13(0.5) | 117(5.6) |  | 13(1.0) | 1(0.1) |  |
| **Statins, n(%)** | | 167(6.3) | 258(12.3) | 0.20933 | 102(8.1) | 91(7.2) | 0.03279 |
| **Oral hypoglycemic agents, n(%)** | | 174(6.5) | 167(8.0) | 0.05556 | 83(6.6) | 83(6.6) | 0.00000 |
| **Insulin preparations, n(%)** | | 7(0.3) | 24(1.1) | 0.10568 | 7(0.6) | 6(0.5) | 0.01107 |
| **Anticoagulants, n(%)** | | 3(0.1) | 32(1.5) | 0.1573 | 3(0.2) | 1(0.1) | 0.03983 |
| **Antiplatelets, n(%)** | | 12(0.4) | 79(3.8) | 0.23245 | 10(0.8) | 5(0.4) | 0.05154 |
| **Nitrates, n(%)** | | 22(0.8) | 75(3.6) | 0.18839 | 18(1.4) | 15(1.2) | 0.02092 |
| **Other lipid-lowering agents, n(%)** | | 38(1.4) | 13(0.6) | 0.08008 | 12(1.0) | 12(1.0) | 0.00000 |
| **Aspirin, n(%)** | | 96(3.6) | 190(9.1) | 0.22569 | 62(4.9) | 55(4.4) | 0.02637 |
| **Other NSAIDs, n(%)** | | 189(7.1) | 150(7.2) | 0.00258 | 94(7.4) | 90(7.1) | 0.01219 |
| **Coxibs, n(%)** | | 2(0.1) | 16(0.8) | 0.10666 | 2(0.2) | 1(0.1) | 0.02299 |
| **Digoxin, n(%)** | | 8(0.3) | 13(0.6) | 0.04730 | 4(0.3) | 4(0.3) | <0.00001 |

Abbreviations: propensity score, propensity score; SMD, standard mean difference; URBMI, Urban Residents Basic Health Insurance, UEBMI, Urban Employee Basic Health Insurance; NRCMS, New Rural Cooperative Medical Scheme; ED, emergency department; NSAIDs, nonsteroidal anti-inflammatory drugs.

## Sensitive analysis

### Subgroup analysis

#### Age group


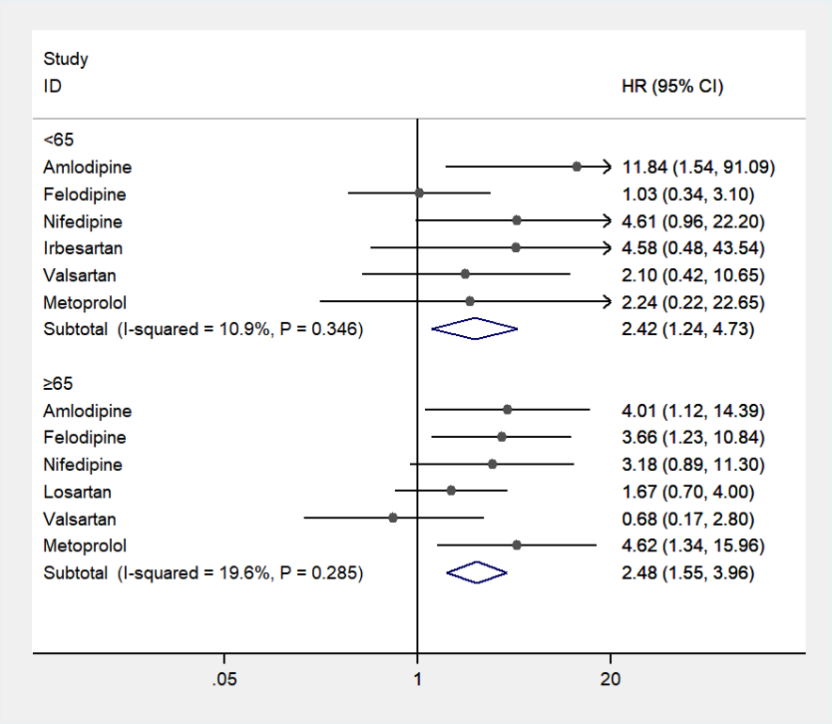


**Figure1** Estimated HR and 95% CI of hospitalization for hypertension-related CVD of originator initiators vs generic initiators in different age groups (patients aged < 65 and patients aged ≥ 65). Note: There was no hospitalization event for hypertension-related CVD of losartan generic initiators aged < 65 and irbesartan generic initiators aged ≥ 65. Abbreviations: HR, hazard ratio; CI, confidence interval; CVD, cardiovascular diseases.

#### Patients without prior hospitalization and ED visits, patients without prior MI, stroke, and CHF, and patients without treatment discontinuation within 180 days at the beginning of follow-up

**Table10** Subgroup analysis of hospitalization for hypertension-related CVD of originator vs generic initiators after 1:1 propensity score matching.

| **Drug cohort** | | **Patients without prior hospitalization and ED visits** | **Patients without prior MI, stroke, and CHF** | **Patients without treatment discontinuation within 180 days** |
| --- | --- | --- | --- | --- |
|  |  | HR(95%CI) | HR(95%CI) | HR(95%CI) |
| **CCBs** | **Amlodipine** | 5.5(1.87, 16.19) | 3.19(1.31, 7.75) | 4.41(1.69, 11.54) |
|  | **Felodipine** | 2.43(1.14, 5.17) | 2.27(1.28, 4.04) | 2.27(1.05, 4.91) |
|  | **Nifedipine** | 2.91(1.06, 7.99) | 6.81(1.57, 29.53) | 1.89(0.78, 4.61) |
| **ARBs** | **Irbesartan** | 2.76(0.76, 9.95) | 3.08(0.83, 11.53) | 2.57(1.39, 4.74) |
|  | **Losartan** | 1.6(0.71, 3.62) | 1.59(0.71, 3.55) | 3.85(1.3, 11.39) |
|  | **Valsartan** | 1.12(0.42, 3.02) | 2.04(0.64, 6.52) | 1.64(0.58, 4.68) |
| **Beta-blocker** | **Metoprolol** | 2.11(0.74, 5.99) | 1.76(0.7, 4.44) | 2.53(0.94, 6.81) |

Abbreviations: ED, emergency department; MI, myocardial infarction; CHF, congestive heart failure; CVD, cardiovascular diseases; propensity score, propensity score; CCB, calcium channel blocker; ARB, angiotensin receptor blocker.

### Different incubation and latent time

#### Hospitalization for hypertension-related CVD

**Table11** Estimated HR and 95% CI of hospitalization for hypertension-related CVD of originator vs generic initiators after 1:1 propensity score matching with different latent and incubation time.

| **Drugs** | **Latent/days**  **Incubation/days** | **0** | **30** | **60** | **90** |
| --- | --- | --- | --- | --- | --- |
|  |  | **HR(95% CI)** | **HR(95% CI)** | **HR(95% CI)** | **HR(95% CI)** |
| **CCBs** | **Amlodipine** | | | | |
|  | **0** | 2.81(1.55, 5.11) | 2.71(1.52, 4.83) | 2.71(1.52, 4.84) | 2.72(1.52, 4.85) |
|  | **30** | 3.04(1.47, 6.26) | 3.13(1.52, 6.42) | 3.11(1.52, 6.38) | 3.48(1.65, 7.36) |
|  | **60** | 3.21(1.49, 6.91) | 3.20(1.49, 6.91) | 3.21(1.49, 6.91) | 2.92(1.40, 6.11) |
|  | **90** | 3.18(1.43, 7.11) | 3.64(1.56, 8.46) | 3.55(1.66, 7.57) | 3.18(1.43, 7.11) |
|  | **Felodipine** | | | | |
|  | **0** | 2.69(1.56, 4.64) | 2.68(1.55, 4.62) | 2.61(1.53, 4.45) | 2.47(1.46, 4.17) |
|  | **30** | 2.57(1.36, 4.97) | 2.23(1.20, 4.17) | 2.31(1.24, 4.31) | 2.49(1.32, 4.71) |
|  | **60** | 2.68(1.31, 5.45) | 2.92(1.40, 6.09) | 2.36(1.22, 4.60) | 2.37(1.22, 4.60) |
|  | **90** | 3.60(1.63, 7.98) | 3.60(1.63, 7.99) | 2.89(1.39, 6.02) | 2.36(1.24, 4.47) |
|  | **Nifedipine** | | | | |
|  | **0** | 2.85(1.60, 5.06) | 3.03(1.68, 5.46) | 3.02(1.67, 5.44) | 2.84(1.59, 5.05) |
|  | **30** | 2.82(1.25, 6.36) | 2.81(1.25, 6.33) | 2.83(1.26, 6.37) | 2.82(1.25, 6.34) |
|  | **60** | 2.68(1.13, 6.37) | 2.68(1.13, 6.36) | 2.35(0.98, 5.66) | 2.67(1.12, 6.33) |
|  | **90** | 3.86(1.26, 11.81) | 3.91(1.21, 11.95) | 3.90(1.28, 11.91) | 3.86(1.26, 11.8) |
| **ARBs** | **Irbesartan** | | | | |
|  | **0** | 1.76(0.79, 3.95) | 1.62(0.74, 3.53) | 1.77(0.79, 3.97) | 1.60(0.74, 3.5) |
|  | **30** | 2.07(0.77, 5.57) | 2.50(0.88, 7.08) | 2.10(0.79, 5.58) | 1.40(3.35, 3.35) |
|  | **60** | 3.81(1.07, 13.5) | 4.03(1.14, 14.28) | 3.99(1.12, 14.12) | 2.23(0.78, 6.32) |
|  | **90** | 1.19(0.50, 2.84 ) | 1.30(0.52, 3.21) | 1.34(0.54, 3.35) | 1.43(0.55, 3.71) |
|  | **Losartan** | | | | |
|  | **0** | 2.84(1.60, 5.04) | 2.85(1.61, 5.05) | 3.03(1.68, 5.45) | 2.70(1.54, 4.73) |
|  | **30** | 2.01(1.05, 3.84) | 2.32(1.17, 4.61) | 2.56(1.26, 5.20) | 2.02(1.05, 3.86) |
|  | **60** | 3.32(1.43, 7.70) | 2.58(1.20, 5.56) | 2.90(1.30, 6.46) | 2.34(1.12, 4.92) |
|  | **90** | 1.85(0.84, 4.07) | 1.81(0.82, 3.99) | 1.65(0.77, 3.55) | 1.51(0.72, 3.18) |
|  | **Valsartan** | | | | |
|  | **0** | 2.57(1.28, 5.14) | 2.37(1.22, 4.63) | 2.82(1.38, 5.77) | 2.57(1.29, 5.12) |
|  | **30** | 1.30(0.64, 2.65) | 1.53(0.73, 3.20) | 1.41(0.69, 2.91) | 1.42(0.69, 2.93) |
|  | **60** | 1.20(0.54, 2.71) | 1.34(0.58, 3.10) | 1.21(0.53, 2.72) | 1.31(0.57, 3.05) |
|  | **90** | 2.04(0.72, 5.78) | 2.08(0.73, 5.88) | 2.06(0.73, 5.82) | 2.07(0.73, 5.87) |
| **Beta-blocker** | **Metoprolol** | | | | |
|  | **0** | 1.57(0.91, 2.72) | 1.73(0.99, 3.02) | 1.79(1.01, 3.17) | 1.74(0.99, 3.05) |
|  | **30** | 1.66(0.77, 3.58) | 1.53(0.73, 3.22) | 1.40(0.67, 2.91) | 1.53(0.73, 3.23) |
|  | **60** | 1.57(0.68, 5.96) | 1.41(0.63, 3.16) | 1.14(0.49, 2.27) | 1.13(0.49, 2.61) |
|  | **90** | 1.25(0.56, 2.80) | 1.13(0.51, 2.48) | 1.13(0.51, 2.48) | 1.16(0.53, 2.56) |

Abbreviations: HR, hazard ratio; propensity score, propensity score; CI confidence interval; CVD, cardiovascular diseases; CCB, calcium channel blocker; ARB, angiotensin receptor blocker.

#### Annual hypertension-related medical cost

**Table12** Annual hypertension-related medical cost for originator vs generic initiators after 1:1 propensity score matching with different latent and incubation time.

| **Drug** | **Latent/days** | | | **0** | | **30** | | **60** | | **90** | |
| --- | --- | --- | --- | --- | --- | --- | --- | --- | --- | --- | --- |
|  | **Incubation/days** | **Group** | **N** | **Median(IQR)/RMB** | ***P* value** | **Median(IQR)/RMB** | ***P* value** | **Median(IQR)/RMB** | ***P* value** | **Median(IQR)/RMB** | ***P* value** |
| **CCBs** | **Amlodipine** | | | | | | | | | | |
|  | **0** | **Originator** | 2119 | 1716.7(796.2, 3612.3) | <0.001 | 1716.7(796.2, 3612.3) | <0.001 | 1716.7(796.2, 3608.7) | <0.001 | 1716.7(796.2, 3608.7) | <0.001 |
|  |  | **Generic** | 2112 | 993.5(478.5, 2081.9) |  | 986.2(474.8, 2063.7) |  | 989.8(478.5, 2092.9) |  | 989.8(482.1, 2103.8) |  |
|  | **30** | **Originator** | 1865 | 1457.3(715.9, 2633.5) | <0.001 | 1457.3(715.9, 2633.5) | <0.001 | 715.9(715.9, 2633.5) | <0.001 | 1457.3(715.9, 2633.5) | <0.001 |
|  |  | **Generic** | 1836 | 880.3(412.7, 1702.1) |  | 880.3(416.4, 1709.4) |  | 876.6(412.7, 1702.1) |  | 880.3(420, 1698.4) |  |
|  | **60** | **Originator** | 1705 | 1347.8(642.8, 2352.2) | <0.001 | 1347.8(642.8, 2352.2) | <0.001 | 1347.8(642.8, 2352.2) | <0.001 | 1347.8(642.8, 2352.2) | <0.001 |
|  |  | **Generic** | 1675 | 803.6(376.2, 1504.8) |  | 796.2(368.9, 796.2) |  | 796.2(376.2, 1501.2) |  | 792.6(383.5, 1493.9) |  |
|  | **90** | **Originator** | 1609 | 1307.6(631.9, 2275.5) | <0.001 | 1307.6(631.9, 2275.5) | <0.001 | 1307.6(628.2, 2275.5) | <0.001 | 1307.6(631.9, 2275.5) | <0.001 |
|  |  | **Generic** | 1592 | 759.7(350.6, 1450) |  | 759.7(354.3, 1453.7) |  | 759.7(350.6, 1453.7) |  | 759.7(350.6, 1450) |  |
|  | **Felodipine** | | | | | | | | | | |
|  | **0** | **Originator** | 2622 | 1267.4(573.4, 2812.4) | <0.001 | 1267.4(573.4, 2808.8) | <0.001 | 1260.1(573.4, 2805.1) | <0.001 | 1260.1(573.4, 2805.1) | <0.001 |
|  |  | **Generic** | 2642 | 748.8(328.7, 1705.7) |  | 741.5(328.7, 1683.8) |  | 730.5(317.8, 1683.8) |  | 734.2(321.4, 1683.8) |  |
|  | **30** | **Originator** | 2351 | 1110.4(511.4, 2100.2) | <0.001 | 1110.4(511.4, 2100.2) | <0.001 | 1110.4(511.4, 2096.5) | <0.001 | 1110.4(511.4, 2096.5) | <0.001 |
|  |  | **Generic** | 2362 | 653.8(299.5, 1307.6) |  | 650.1(295.9, 1303.9) |  | 650.1(295.9, 1303.9) |  | 653.8(299.5, 1300.3) |  |
|  | **60** | **Originator** | 2159 | 1022.7(493.1, 1851.8) | <0.001 | 1022.7(493.1, 1851.8) | <0.001 | 1026.4(493.1, 1851.8) | <0.001 | 1026.4(493.1, 1851.8) | <0.001 |
|  |  | **Generic** | 2160 | 639.2(292.2, 1223.6) |  | 624.6(292.2, 1219.9) |  | 595.4(273.9, 1176.1) |  | 602.7(277.6, 1176.1) |  |
|  | **90** | **Originator** | 2055 | 982.5(460.2, 1760.5) | <0.001 | 982.5(460.2, 1760.5) | <0.001 | 982.5(460.2, 1760.5) | <0.001 | 982.5(456.6, 1756.9) | <0.001 |
|  |  | **Generic** | 2059 | 569.8(263, 1110.4) |  | 573.4(263, 1117.7) |  | 573.4(263, 1117.7) |  | 577.1(255.7, 1110.4) |  |
|  | **Nifedipine** | | | | | | | | | | |
|  | **0** | **Originator** | 1238 | 2019.8(829.1, 4978.4) | <0.001 | 2019.8(829.1, 4978.4) | <0.001 | 2019.8(829.1, 4978.4) | <0.001 | 2019.8(829.1, 4978.4) | <0.001 |
|  |  | **Generic** | 1265 | 610.0(222.8, 1621.7) |  | 610.0(226.5, 1629.0) |  | 610.0(222.8, 1643.6) |  | 613.6(222.8, 1643.6) |  |
|  | **30** | **Originator** | 1043 | 1472.0(631.9, 2735.7) | <0.001 | 1472.0(631.9, 2735.7) | <0.001 | 1472.0(631.9, 2735.7) | <0.001 | 1472.0(631.9, 2735.7) | <0.001 |
|  |  | **Generic** | 1065 | 456.6(189.9, 1183.4) |  | 460.2(193.6, 1205.3) |  | 460.2(193.6, 1183.4) |  | 471.2(200.9, 1205.3) |  |
|  | **60** | **Originator** | 1098 | 1314.9(610.0, 2443.5) | <0.001 | 1314.9(610.0, 2443.5) | <0.001 | 1314.9(610.0, 2443.5) | <0.001 | 1314.9(610.0, 2443.5) | <0.001 |
|  |  | **Generic** | 1153 | 442.0(186.3, 1030.0) |  | 442.0(189.9, 1033.7) |  | 442.0(189.9, 1030.0) |  | 442.0(189.9, 1030.0) |  |
|  | **90** | **Originator** | 883 | 1260.1(573.4, 2333.9) | <0.001 | 1260.1(573.4, 2333.9) | <0.001 | 1260.1(573.4, 2333.9) | <0.001 | 1260.1(573.4, 2333.9) | <0.001 |
|  |  | **Generic** | 895 | 420.0(171.7, 986.2) |  | 420.0(171.7, 986.2) |  | 416.4(171.7, 978.9) |  | 416.4(171.7, 971.6) |  |
| **ARBs** | **Irbesartan** | | | | | | | | | | |
|  | **0** | **Originator** | 717 | 1833.6(818.2, 3849.7) | <0.001 | 1833.6(818.2, 3849.7) | <0.001 | 1833.6(818.2, 3849.7) | <0.001 | 1833.6(818.2, 3849.7) | <0.001 |
|  |  | **Generic** | 698 | 978.9(467.5, 2071) |  | 1030.0(482.1, 2129.4) |  | 964.3(456.6, 2034.4) |  | 1051.9(496.7, 2231.7) |  |
|  | **30** | **Originator** | 653 | 1596.1(770.7, 3115.6) | <0.001 | 1596.1(770.7, 3115.6) | <0.001 | 1596.1(770.7, 3115.6) | <0.001 | 1596.1(770.7, 3115.6) | <0.001 |
|  |  | **Generic** | 632 | 902.2(412.7, 1873.7) |  | 913.1(412.7, 1870.1) |  | 946.0(431.0, 1877.4) |  | 949.7(438.3, 2005.2) |  |
|  | **60** | **Originator** | 601 | 1512.1(737.8, 2914.7) | <0.001 | 1512.1(737.8, 2914.7) | <0.001 | 1512.1(737.8, 2914.7) | <0.001 | 1512.1(737.8, 2914.7) | <0.001 |
|  |  | **Generic** | 582 | 843.7(405.4, 1683.8) |  | 832.8(405.4, 1683.8) |  | 836.4(394.5, 1636.3) |  | 829.1(423.7, 1632.7) |  |
|  | **90** | **Originator** | 575 | 1472(704.9, 2801.5) | <0.001 | 1472.0(704.9, 2801.5) | <0.001 | 1472(704.9, 2801.5) | <0.001 | 1472(704.9, 2801.5) | <0.001 |
|  |  | **Generic** | 557 | 836.4(390.8, 1687.5) |  | 814.5(390.8, 1629.0) |  | 788.9(376.2, 1614.4) |  | 756.1(383.5, 1534.1) |  |
|  | **Losartan** | | | | | | | | | | |
|  | **0** | **Originator** | 1960 | 2081.9(1004.4, 4554.7) | <0.001 | 2081.9(1004.4, 4554.7) | <0.001 | 2081.9(1004.4, 4554.7) | <0.001 | 2081.9(1004.4, 4554.7) | <0.001 |
|  |  | **Generic** | 2061 | 1643.6(810.9, 3374.9) |  | 1661.9(810.9, 3426.0) |  | 1614.4(788.9, 3327.4) |  | 1618.1(796.2, 3360.3) |  |
|  | **30** | **Originator** | 1704 | 1797.0(883.9, 3269.0) | <0.001 | 1797.0(883.9, 3269.0) | <0.001 | 1797.0(883.9, 3269.0) | <0.001 | 1797(883.9, 3269.0) | <0.001 |
|  |  | **Generic** | 1775 | 1417.2(723.2, 2607.9) |  | 1420.8(723.2, 2622.5) |  | 1417.2(723.2, 2600.6) |  | 1413.5(730.5, 2629.8) |  |
|  | **60** | **Originator** | 1548 | 1676.5(836.4, 2976.8) | <0.001 | 1676.5(836.4, 2976.8) | <0.001 | 1676.5(836.4, 2976.8) | <0.001 | 1676.5(836.4, 2976.8) | <0.001 |
|  |  | **Generic** | 1612 | 1300.3(650.1, 2363.2) |  | 1285.7(635.5, 2355.9) |  | 1293.0(639.2, 2341.3) |  | 1289.3(639.2, 2326.6) |  |
|  | **90** | **Originator** | 1460 | 1596.1(814.5, 2816.1) | <0.001 | 1596.1(814.5, 2816.1) | <0.001 | 1596.1(814.5, 2816.1) | <0.001 | 1596.1(814.5, 2816.1) | <0.001 |
|  |  | **Generic** | 1519 | 1205.3(599, 2184.2) |  | 1216.3(624.6, 2206.1) |  | 1201.7(610.0, 2206.1) |  | 1201.7(599.0, 2184.2) |  |
|  | **Valsartan** | | | | | | | | | | |
|  | **0** | **Originator** | 2443 | 5668.7(905.8, 3568.5) | <0.001 | 1815.3(905.8, 3568.5) | <0.001 | 1815.3(905.8, 3568.5) | <0.001 | 1815.3(905.8, 3568.5) | <0.001 |
|  |  | **Generic** | 2614 | 2348.6(540.6, 2092.9) |  | 1070.2(540.6, 2103.8) |  | 1062.9(544.2, 2144) |  | 1070.2(544.2, 2103.8) |  |
|  | **30** | **Originator** | 2167 | 1588.8(818.2, 2779.6) | <0.001 | 1588.8(818.2, 2779.6) | <0.001 | 1588.8(818.2, 2779.6) | <0.001 | 1588.8(818.2, 2779.6) | <0.001 |
|  |  | **Generic** | 2314 | 960.6(482.1, 1808.0) |  | 960.6(485.8, 1833.6) |  | 964.3(489.4, 1815.3) |  | 957.0(478.5, 1837.2) |  |
|  | **60** | **Originator** | 2016 | 1501.2(763.4, 2523.9) | <0.001 | 1501.2(763.4, 2523.9) | <0.001 | 1501.2(763.4, 2523.9) | <0.001 | 1501.2(763.4, 2523.9) | <0.001 |
|  |  | **Generic** | 2128 | 909.5(467.5, 1680.2) |  | 913.1(467.5, 1680.2) |  | 913.1(471.2, 1661.9) |  | 913.1(463.9, 1672.8) |  |
|  | **90** | **Originator** | 1924 | 1417.2(737.8, 2432.6) | <0.001 | 1417.2(737.8, 2432.6) | <0.001 | 1417.2(737.8, 2432.6) | <0.001 | 1417.2(737.8, 2432.6) | <0.001 |
|  |  | **Generic** | 2019 | 862.0(438.3, 1585.2) |  | 862.0(438.3, 1588.8) |  | 858.3(434.6, 1607.1) |  | 858.3(438.3, 1596.1) |  |
| **Beta-blocker** | **Metoprolol** | | | | | | | | | | |
|  | **0** | **Originator** | 1679 | 1073.8(379.9, 2947.6) | 0.001 | 1073.8(379.9, 2954.9) | <0.001 | 1073.8(379.9, 2951.2) | <0.001 | 1073.8(383.5, 2943.9) | <0.001 |
|  |  | **Generic** | 1724 | 957.0(310.5, 2144.0) |  | 975.2(325.1, 2158.6) |  | 964.3(314.1, 2140.4) |  | 957.0(325.1, 2144.0) |  |
|  | **30** | **Originator** | 1481 | 847.4(314.1, 1939.5) | 0.389 | 851.0(314.1, 1943.1) | 0.285 | 847.4(314.1, 1939.5) | 0.548 | 847.4(310.5, 1932.2) | 0.375 |
|  |  | **Generic** | 1501 | 825.5(284.9, 1826.3) |  | 840.1(288.5, 1826.3) |  | 825.5(277.6, 1815.3) |  | 821.8(284.9, 1804.3) |  |
|  | **60** | **Originator** | 1324 | 759.7(284.9, 1694.8) | 0.456 | 759.7(284.9, 1694.8) | 0.600 | 752.4(284.9, 1698.4) | 0.375 | 756.1(284.9, 1698.4) | 0.406 |
|  |  | **Generic** | 1340 | 719.5(263, 1654.6) |  | 726.8(266.6, 1658.2) |  | 759.7(266.6, 1661.9) |  | 767.0(266.6, 1661.9) |  |
|  | **90** | **Originator** | 1230 | 704.9(263.0, 1530.4) | 0.646 | 704.9(263.0, 1530.4) | 0.473 | 701.3(266.6, 1512.1) | 0.863 | 704.9(266.6, 1512.1) | 1.000 |
|  |  | **Generic** | 1248 | 741.5(266.6, 1647.3) |  | 737.8(263.0, 1643.6) |  | 741.5(263.0, 1643.6) |  | 737.8(263.0, 1640.0) |  |

Abbreviations: propensity score, propensity score; IQR, interquartile range; CCB, calcium channel blocker; ARB, angiotensin receptor blocker.

### Estimated impact of income on the initiation of originator products or generics

**Table13** Estimated impact of income on the patient initiation of originator products vs generics.

| **Sample Characteristics** | **OR** | **95%CI** |
| --- | --- | --- |
| Income/year |  |  |
| ＜RMB2500 | Reference | Reference |
| RMB2500-5000 | 2.25 | (0.87, 5.80) |
| RMB5000-7500 | 0.96 | (0.26, 3.59) |
| ＞ RMB7500 | 2.03 | (0.79, 5.21) |

Abbreviations: OR, odds ratio; CI, confidence interval.

Note: Logistic regression was used to estimate the impact of income on the patient initiation of originator products vs generics, adjusted by baseline covariates.

### Secondary outcome


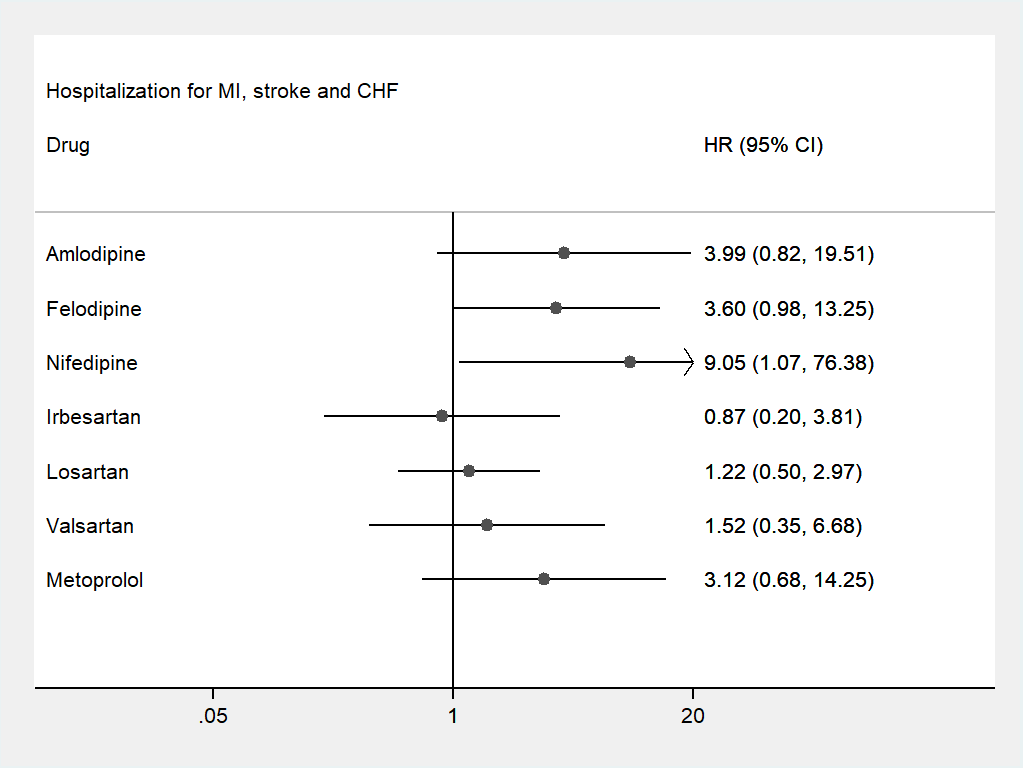


**Figure2** Estimated HR and 95% CI of hospitalization for hypertension-related MI, stroke, and CHF of originator initiators vs generic initiators after 1:1 propensity score matching. Abbreviations: HR, hazard ratio; CI, confidence interval; MI, myocardial infarction; CHF, congestive heart failure; propensity score, propensity score.
